# Smek promotes corticogenesis through regulating Mbd3’s stability and Mbd3/NuRD complex recruitment to genes associated with neurogenesis

**DOI:** 10.1371/journal.pbio.2001220

**Published:** 2017-05-03

**Authors:** Byoung-San Moon, Hyung-Mun Yun, Wen-Hsuan Chang, Bradford H. Steele, Mingyang Cai, Si Ho Choi, Wange Lu

**Affiliations:** Broad Center for Regenerative Medicine and Stem Cell Research, Department of Stem Cell Biology and Regenerative Medicine, Keck School of Medicine, University of Southern California, Los Angeles, California, United States of America; California Institute of Technology, United States of America

## Abstract

The fate of neural progenitor cells (NPCs) during corticogenesis is determined by a complex interplay of genetic or epigenetic components, but the underlying mechanism is incompletely understood. Here, we demonstrate that Suppressor of Mek null (Smek) interact with methyl-CpG–binding domain 3 (Mbd3) and the complex plays a critical role in self-renewal and neuronal differentiation of NPCs. We found that Smek promotes Mbd3 polyubiquitylation and degradation, blocking recruitment of the repressive Mbd3/nucleosome remodeling and deacetylase (NuRD) complex at the neurogenesis-associated gene loci, and, as a consequence, increasing acetyl histone H3 activity and cortical neurogenesis. Furthermore, overexpression of Mbd3 significantly blocked neuronal differentiation of NPCs, and Mbd3 depletion rescued neurogenesis defects seen in *Smek1/2* knockout mice. These results reveal a novel molecular mechanism underlying Smek/Mbd3/NuRD axis-mediated control of NPCs’ self-renewal and neuronal differentiation during mammalian corticogenesis.

## Author summary

Neural progenitor cells are self-renewing, multipotent cells that generate major neural cell types, including neurons and glia. Their fate during development of the cerebral cortex is determined by a complex interplay of genetic and epigenetic components. It has been shown that Suppressor of Mek null (Smek) proteins—which are evolutionarily conserved—play a role during the asymmetric cell division of neuroblasts in invertebrates. Methyl-CpG–binding domain 3 (Mbd3) protein, a core component of the repressive nucleosome remodeling and deacetylase (NuRD) complex, is an important epigenetic regulator that plays an essential role in mammalian development. In this study, we discovered that Smek interacts with Mbd3 and promotes its degradation via a posttranslational modification called polyubiquitylation. Degradation of Mb3, in turn, blocks recruitment of Mbd3/NuRD complex on target gene promoters, leading to an increase in neuronal differentiation during cortical development. This study not only elucidates a distinct mechanism for Smek-mediated neuronal differentiation but also identifies Smek as a negative regulator of the Mbd3 protein during cortical brain development.

## Introduction

Neural stem cells (NSCs) are self-renewing, multipotent cells that generate major neural cell types, including neurons and glia, in the developing central nervous system (CNS) [1,2]. During neurogenesis, NSCs are derived from neuroepithelial cells (NECs), which first divide symmetrically to expand the population and then undergo a series of asymmetric cell divisions to produce neural progenitor cells (NPCs), lineage-restricted precursor cells (RPCs), and mature neural cells [3]. NSC fate determination is tightly regulated by intrinsic and extrinsic factors [4–6]. Recent findings suggest that neurodevelopmental and neurological anomalies, such as schizophrenia, autism, and depression, can emerge from abnormal specification, growth, and differentiation of NSCs [6–8].

Suppressor of Mek null (Smek), an evolutionarily conserved protein family, consists of two isoforms, Smek1 (PP4R3A) and Smek2 (PP4R3B), first reported as playing a role in the formation of a functional phosphatase group with PP4c, PP4R1, and PP4R2 complex [9]. Smek was initially identified in *Dictyostelium discoideum* as a playing a role in cell polarity, chemotaxis, and gene expression [10]. Smek also has several functions in lower eukaryotes, such as *Caenorhabditis elegans*, including roles in longevity by modulating DAF-16/FOXO3a transcriptional activity [11], DNA repair through dephosphorylation of phosphorylated H2AX (g-H2AX) during DNA replication [12], and glucose metabolism by controlling cAMP-response element binding protein (CREB)-regulated, transcriptional coactivator 2 (CRTC2)-dependent gene expression [13]. Notably, Smek also plays a critical role in cell-fate determination in higher eukaryotes. In *Drosophila* neuroblasts, PP4R3/Falafel (Flfl), which is an orthologous of Smek and is conserved throughout eukaryotic evolution, regulates asymmetric cell division by controlling localization of Miranda [14–16]. In mice, which express orthologous Smek 1 and 2, both Smek proteins suppress *brachyury* expression in embryonic stem cells (ESCs), and Smek1, especially, promotes NSC neuronal differentiation by negatively regulating Par3 [14–16]. Although we have shown that the Smek isoform Smek1 promotes NSC neuronal differentiation, signaling pathways required for that activity remain unclear [15].

Methyl-CpG–binding domain protein 3 (Mbd3), a core component of the repressive nucleosome remodeling and deacetylase (NuRD) complex, possesses a conserved methyl-CpG–binding domain (Mbd) [17,18]. Unlike other family members, which recognize 5′-methyl-cytosine (5′-mC)-modified DNA, Mbd3 specifically recognizes 5′-hydroxymethyl-cytosine (5′-hmC), an epigenetic marker highly enriched in NSCs [19,20]. Mbd3 plays an important role in brain development. Mbd3 expression is reported to be predominant in cortical NECs of the embryonic forebrain [21]. Mice lacking Mbd3 die in utero before neurogenesis is completed [22]. Conditional knockout of Mbd3 in neural progenitor cells leads to defects of differentiation of appropriate cell types during neurogenesis [23]. Despite emerging evidence that Mbd3 has a critical function in the CNS, little is known about its regulatory mechanism in NSCs.

To understand Smek protein function during mammalian CNS neurogenesis, we screened for novel Smek-binding proteins that regulate NPC neuronal differentiation and identified Mbd3, a potent epigenetic regulator, as a Smek-interacting protein. We found that Mbd3 is highly expressed in NPC populations in the ventricular zone, and it was predominantly expressed in the nucleus. Smek interacted directly with the Mbd3’s Mbd domain, destabilizing Mbd3 protein and its interaction with NuRD components, and sequentially, preventing accumulation of the Mbd3/NuRD complex on target gene loci functioning in neurogenesis. Such dissociation of Mbd3/NuRD complex promotes NPC neuronal differentiation. Moreover, overexpression of Mbd3 significantly inhibited neuronal differentiation of *wild-type* NPCs, while Mbd3 depletion rescued neurogenesis defects seen in *Smek* knockout mice.

This work identifies a novel pathway of Smek and Mbd3/NuRD complex in brain development and could encourage discovery of novel epigenetic regulators governing neuronal differentiation.

## Results

### Double knockout of *Smek1/2* triggers severe neurogenesis defects in vitro and in vivo

**Fig 1:**
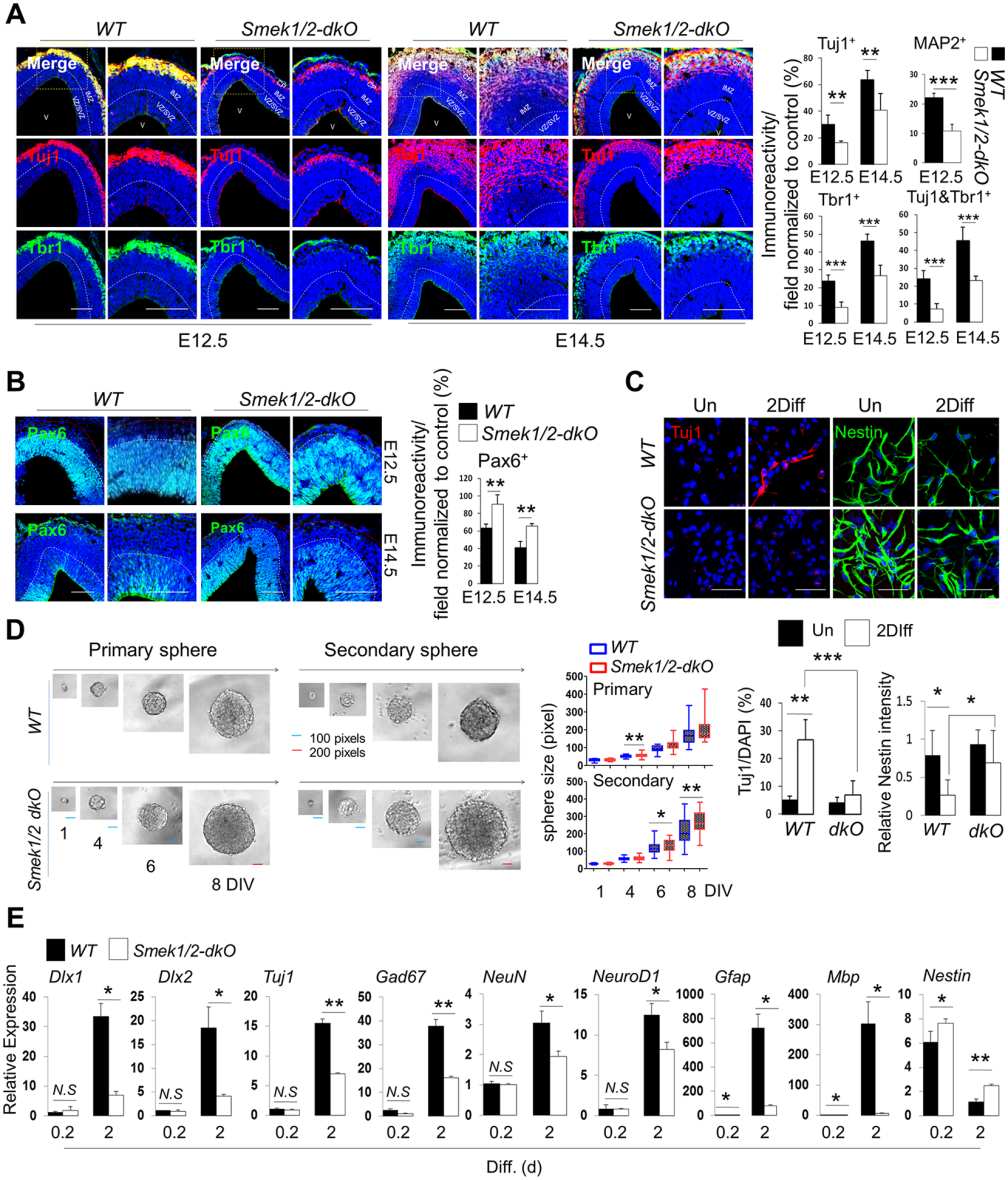
Suppressor of Mek null (Smek) expression and function in mouse cortical development. (A) Fixed cyroembedded coronal sections from E12.5 or E14.5 mouse forebrain stained with antibodies against Tuj1 (red), Tbr1 (green), and microtubule-associated protein 2 (MAP2) (red). Nuclear staining is shown by 4',6-diamidino-2-phenylindole (DAPI) (blue). (Right) Quantification of Fig 1A and S1D Fig. (E12.5: *WT*, *n* = 4, *dKO*, *n* = 5; E14.5: *WT*, *n* = 6, *dKO*, *n* = 5). Scale bars, 50 μm. (B) Same as Fig 1A for Pax6. (Right) Quantification of Fig 1B (E12.5: *WT*, *n* = 5, *dKO*, *n* = 4; E14.5: *WT*, *n* = 6, *dKO*, *n* = 3). Scale bar, 50 μm. (C) Immunostaining with Nestin (green) and Tuj1 (red) antibodies in *WT* and *Smek1/2 dKO* neural progenitor cells (NPCs). Nuclear staining is shown by DAPI (blue). Scale bars, 50 μm. (Lower) Quantification of anti-Tuj1–positive (*WT*-Un, *n* = 3; *WT*-2DIV, *n* = 6; *dKO*-Un, *n* = 3; *dKO*-2DIV, *n* = 6) and anti-Nestin–postive (*WT*-Un, *n* = 6; *WT*-2DIV, *n* = 6; *dKO*-Un, *n* = 6; *dKO*-2DIV, *n* = 6) cells in Fig 1C. Un, undifferentiation; 2Diff, differentiation 2 d in vitro (DIV). (D) Single cells of *WT* and *Smek1/2 dKO* NPCs were separated by serial dilution and sphere formation was induced for 8 d in vitro. Relative size of primary spheres grown up to 8 DIV were quantified by the ImageJ quantification software. Scale bars, blue (100 pixel), red (200 pixel). (E) Quantitative PCR (qPCR) analysis of indicated mRNAs. Values correspond to the average ± SD. Diff. (d), days in differentiation. Statistical *t* test analysis was performed to calculate significance (*p < 0.05, **p < 0.005, ***p < 0.0005; not significant (ns), *p* > 0.05). All quantification data underlying panels A–D can be found in S2 Data.

Recently, we reported that Smek1 promotes neurogenesis during mouse cortical development [15]. To further characterize Smek function in neurogenesis, we generated Smek1 and Smek2 double knockout (dKO) mice and set out to analyze cortical development in *Smek1* knockout (*KO*) and *Smek1* and *Smek2 dKO* embryo brains (S1A and S1B Fig). To do so, we undertook immunohistochemical analysis of the embryonic cortex derived from *WT* and *Smek1/2 dKO* mice and observed a decrease in the number of cells positive for Tuj1, an early neuronal marker (~15% and ~25% fewer Tuj1+ cells at E12.5 and E14.5, respectively) (Fig 1A). We observed similar decreases in postmitotic cortical neuron marker Tbr1-positive cells (~15% and ~20% reductions at E12.5 and E14.5, respectively) and Tuj1 and Tbr1 double-positive cells (near 20% reduction in each stage) (Fig 1A). The number of mature microtubule-associated protein 2 (MAP2)-positive neurons also significantly decreased by ~12% in the E12.5 cortex (Fig 1A, right and S1C Fig). In *Smek1/2* mice, the number of Pax6-positive NPCs increased significantly (by ~23% at both E12.5 and E14.5), as did Nestin/Ki67 double-positive cells (~24% increase at E12.5) compared with *wild-type* (*WT*) mice (Fig 1B and S1D–S1F Fig). As expected, neurogenesis defects in *Smek1/2 dKO* embryonic brains were greater than those seen in *Smek1 KO* mice (S2A and S2B Fig, S1 Table). These results demonstrated that in the *Smek1/2 dKO* mice, the number of neurons is reduced while the number of neural stem cells is increased.

To assess direct effects of Smek loss on NPC differentiation capacity, we cultured *Smek1/2 dKO* NPCs derived from E11.5 mouse embryo brains in the presence of basic fibroblast growth factor (bFGF) and then withdrew bFGF to induce neural differentiation. Consistent with in vivo results, the number of Tuj1-positive cells decreased in *Smek1/2 dKO* cultures while the number of Nestin positive cells increased slightly in *Smek1/2 dKO* cultures over the course of differentiation (Fig 1C and S3 Fig). In addition, we assessed the role of Smek in maintenance of self-renewal activity using the single-cell clonal neural sphere formation assay. *Smek1/2 dKO* NPCs showed higher sphere-forming ability than those derived from *WT* NPCs in both primary and secondary sphere-forming assays (Fig 1D). Further analysis using quantitative PCR (qPCR) showed that *Smek1/2 dKO* cells exhibited decreased expression of *Dlx1*, *Dlx2*, *Tuj1*, *Gad67*, *NeuN*, and *NeuroD1*, as well as other neural differentiation genes such as *Gfap* and *Mbp* (the latter an oligodendrocyte marker), and increased expression of Nestin (Fig 1E and S3 Fig). Severe differentiation defects seen in cortical NPCs lacking both *Smek 1* and *2* suggest that these factors compensate for each other during cortical development. All these experiments demonstrated that Smek plays a role in self-renewal and neural differentiation of NPCs in vivo and in vitro.

### Smek1/2 interact with Mbd3 during cortical neurogenesis

**Fig 2:**
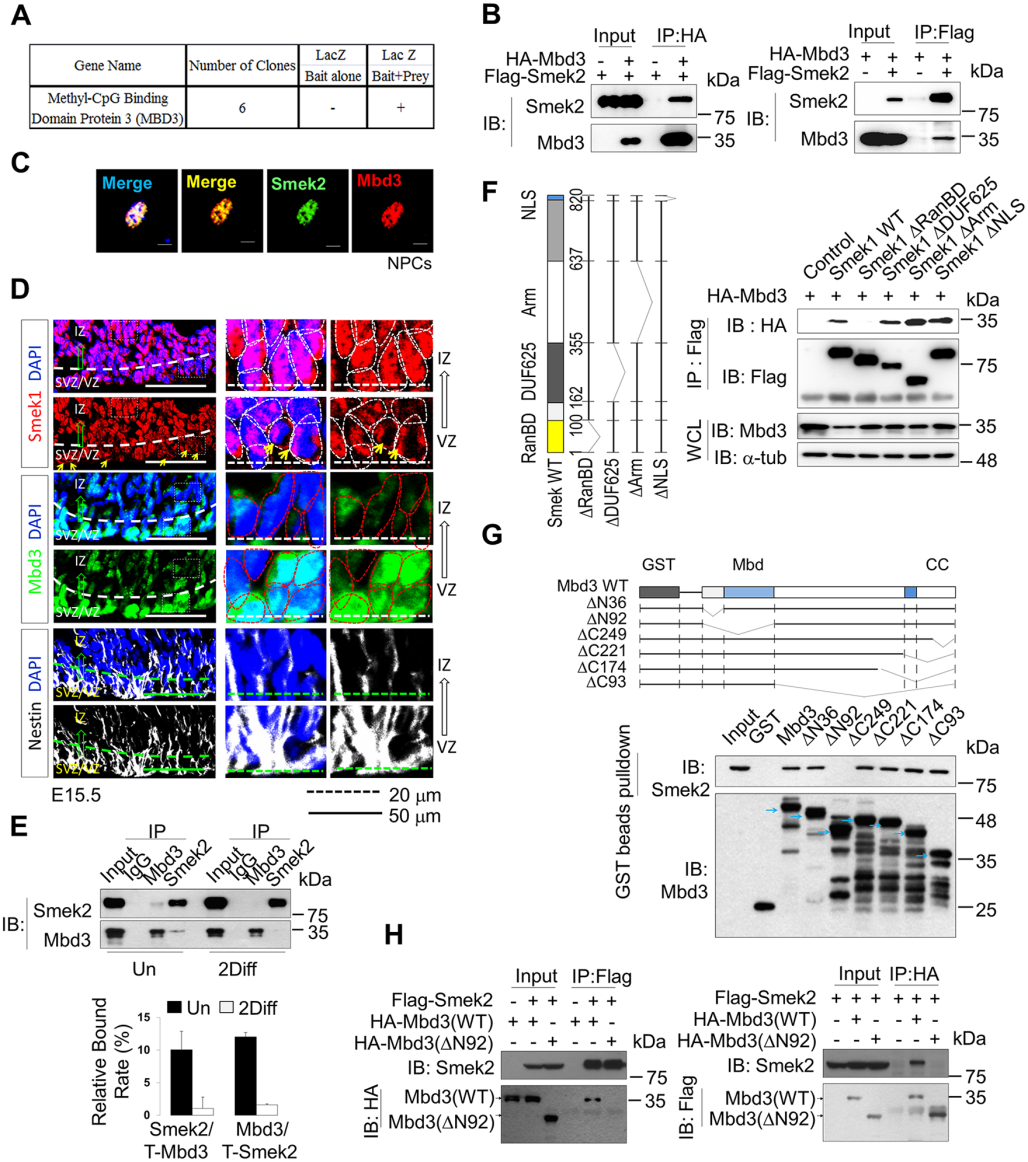
Suppressor of Mek null (Smek) interacts with methyl-CpG–binding domain protein 3 (Mbd3) and both colocalize in the nucleus. (A) Numerical result of clones observed by β-galactosidase colony-lift filter assay using bait (Smek2) and prey (Mbd3). (B) Coimmunoprecipitation (co-IP) of human influenza hemagglutinin (HA)-tagged Mbd3 or Flag-tagged Smek2 in HEK293T cells transfected with Mbd3 and/or Smek (*n* = 2). IB, immunoblot. (C) Immunostaining with Mbd3 (red) and Smek2 (green) antibodies in NPCs. Nuclear staining is shown by 4',6-diamidino-2-phenylindole (DAPI) (blue). Scale bar, 50 μm. (D) Fixed, cyroembedded coronal sections from E15.5 mouse forebrain stained with antibodies against Mbd3 (green), Smek1 (red), and Nestin (white). Nuclear staining is shown by DAPI (blue). Yellow arrows indicate perinuclear localization of Smek1 in ventricular zone progenitor cells. (E) Co-IP of endogenous Smek2 and Mbd3 in undifferentiated (left) and differentiated (right) NPCs using anti- immunoglobulin G (IgG) (negative control), anti-Mbd3, or anti-Smek2 antibodies (*n* = 2). Un, undifferentiation; Diff, differentiation. (F) (Upper) Schematic of *Smek1* mutants used in deletion analysis. (Lower) Co-IP of these Smek1 mutants with HA-tagged Mbd3 in the cells expressing full-length HA-tagged Mbd3 and Flag-tagged deletion Smek1 mutants (*n* = 2). Immunoprecipitation was performed using an anti-Flag antibody and immunoblotting was done using an anti-HA antibody. WCL, whole cell lysate. (G) (Upper) Schematic showing *Mbd3* deletion mutants used in domain mapping analysis. (Lower) Glutathione-S-transferase (GST) pull-down assay using of purified GST, GST-Mbd3, or deletion mutants that were mixed with Smek2-overexpressing cell lysate. Immunoblotting was performed using Smek2 and Mbd3 antibodies. Blue arrows indicate major bands of each GST-fused Mbd3 mutant protein expression. (H) Co-IP of Smek with full-length or mutant Mbd3 (*n* = 1). Black arrows indicate band of each full-length or mutant (ΔN92) Mbd3. All quantification data underlying panel A can be found in S2 Table and quantification data for panel E can be found in S2 Data.

In order to determine a detailed molecular mechanism modulating the differentiation of NPCs by Smek, we sought to identify proteins interacting with Smek protein. To identify how Smek mediates neuronal differentiation of NPCs, a yeast two-hybrid (Y2H) screening assay was performed and revealed that Smek interacts with full-length Mbd3 (Fig 2A and S2 Table). An immunoprecipitation (IP) assay confirmed the interaction of Smek1 and Smek2 with Mbd3 in 293T cells (Fig 2B, S4A and S4B Fig). As shown in Fig 2B and S4B Fig, Smek1 or Smek2 coimmunoprecipitate with Mbd3, while no signals were detected in cells transfected with a negative control vector. Furthermore, colocalization of endogenous Mbd3 and Smek2 protein was also observed in the nucleus of in vitro cultured NPCs (Fig 2C). Both Smek1 and Mbd3 proteins were expressed in NPCs of the ventricular zone (VZ) and subventricular zone (SVZ) in vivo (Fig 2D, S4C and S4D Fig), and merged images reveal that Smek and Mbd3 staining was nuclear (Fig 2C and 2D). Mbd3 is highly enriched in the nucleus of the VZ progenitor cells and its expression in the nucleus showed a gradually decreasing pattern in the direction of the intermediate zone (IZ) and cortical plate (CP). Smek1 is expressed in the nuclear or perinuclear region of cortical progenitor cells in the VZ, but its expression and nuclear localization is significantly increased in the differentiated neurons in the IZ and CP (Fig 2D, S4C and S4D Fig). The interaction of endogenous Smek and Mbd3 were further confirmed by co-IP of Smek and Mbd3 using NPC lysates. Smek/Mbd3 interaction, however, was apparently disrupted during neuronal differentiation of *wild-type* NPCs, suggesting that protein complexes may function in NPC differentiation (Fig 2E and S4C Fig).

To map Smek and Mbd3 domain(s) required for interaction, we generated Smek1 and Mbd3 mutants and assessed their interaction (Fig 2F–2H). A Smek *N*-terminal deletion mutant (ΔRanBD) did not interact with Mbd3 protein, shown by co-IP of Smek and Mbd3 in HEK293T cells, whereas interaction of other Smek deletion mutants with Mbd3 was comparable to the full-length protein, suggesting the RanBD domain of Smek mediates Smek’s interaction with Mbd3 (Fig 2F). To map to the domains on Mbd3 required for Smek interaction, we generated glutathione-S-transferase (GST) fusion Mbd3 mutant proteins in bacteria and incubated these proteins with HEK293T cell lysates expressing Smek2. GST pull-down assays revealed that the Smek-Mbd3 interaction was disrupted in Mbd3 deletion mutants lacking the first 92 *N*-terminal amino acids (ΔN92), a region encompassing the Mbd domain (Fig 2G, upper panel). This finding was confirmed by IP experiments in HEK293T cells transfected with full-length or ΔN92 forms of Mbd3 and Smek (Fig 2H). Those results indicated that the Mbd domain of Mbd3 is required for Smek interaction.

### Mbd3 protein is destabilized during NPC differentiation

**Fig 3:**
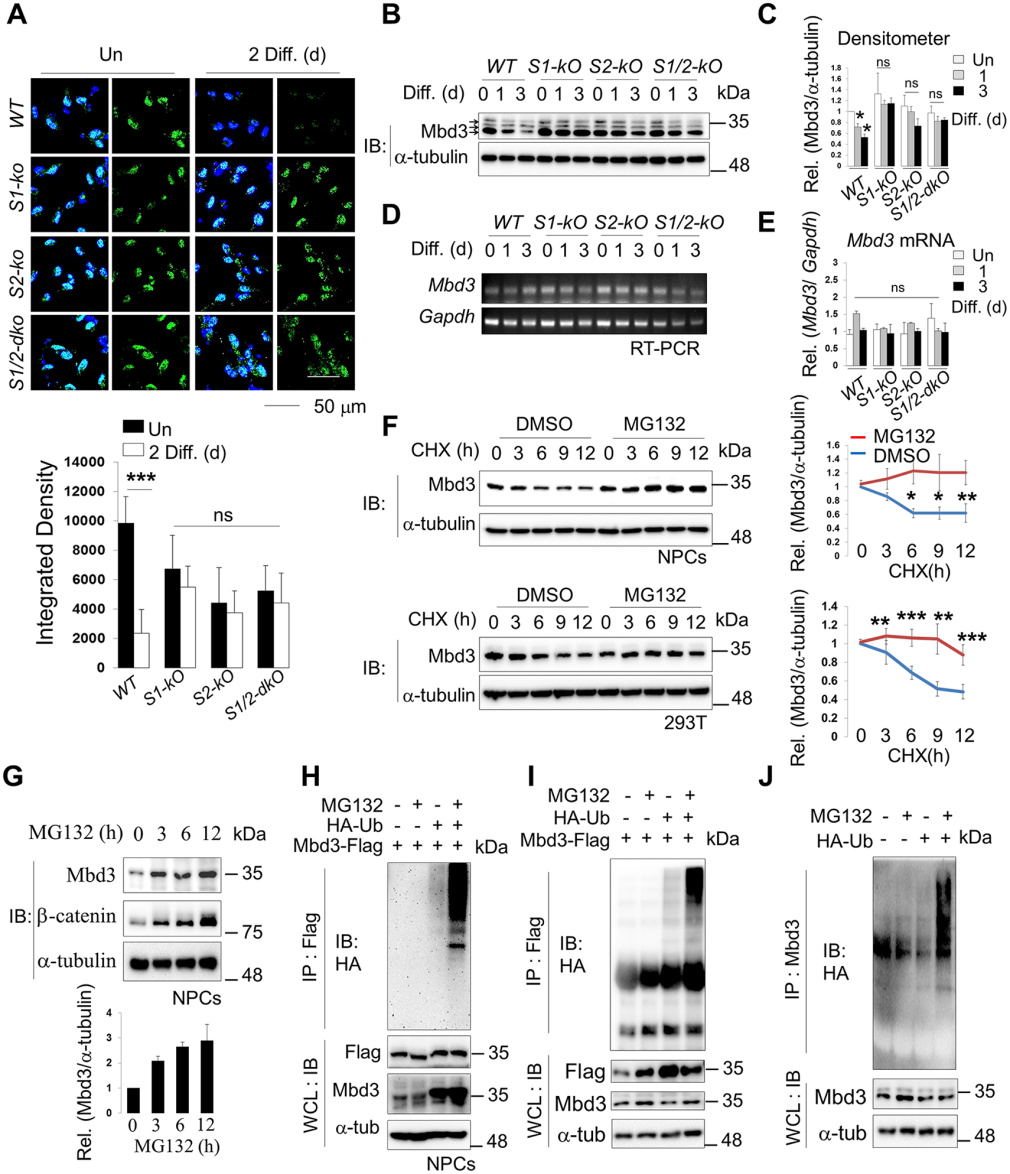
Both endogenous and overexpressed methyl-CpG–binding domain protein 3 (Mbd3) are degraded as neural progenitor cells (NPCs) differentiate. (A) Immunostaining to detect Mbd3 expression in *wild-type* (*WT*), *Smek1 knockout* (*S1-KO*), *Smek2 knockout* (*S2-KO*) and *Smek1/2 double knockout* (*S1/2-dKO*) cells. Nuclear staining is shown by 4',6-diamidino-2-phenylindole (DAPI) (blue). Mbd3 is shown in green. Scale bar, 50 μm. (Lower) Quantification of Fig 3A. Scale bar, 50 μm. Diff. (d), differentiation days in vitro. (B) Western blot of Mbd3 in *WT* or *Smek knockout* NPCs that are under differentiation for 0, 1, or 3 d (*n* = 3). α-tubulin was used as an internal loading control. Arrows indicate multiple Mbd3 isoforms around 30–40 kDa (upper, Mbd3A; middle, Mbd3B; bottom, Mbd3C). (C) Quantification of Fig 3B. (D) Reverse transcription PCR (RT-PCR) analysis of *Mbd3* and *Gapdh* transcript levels at indicated days (d) of differentiation (*n* = 2). (E) Quantification of data shown in Fig 3D. (F) (left panel) Immunoblot (IB) analysis of Mbd3 and α-tubulin in cells treated with cycloheximide (CHX) for indicated times (h, hours) with or without MG132 in NPCs (*n* = 3) or 293T cells (*n* = 5). (Right panel) Quantification of band intensity in Fig 3F, left panel using the ImageJ software. (G) NPCs were treated with MG132 for 6 h, harvested, and the lysates were immunoblotted with anti-Mbd3, anti–α-tubulin and anti–β-catenin antibodies. β-catenin was used as a positive control (*n* = 2). Quantification of band intensity in Fig 3G, bottom panel using the ImageJ software. (H) Ubiquitylation of overexpressed Mbd3 in NPCs (*n* > 3). (I) Ubiquitylation of overexpressed Mbd3 in HEK293T transfected with Flag-Mbd3 and HA-Ub expression vectors and treated 1 d later with MG132 were detected by immunoprecipitation with Flag antibody followed by immunoblotting with HA antibody (upper panel) (*n* > 2). Levels of each protein in the whole cell lysate are shown with western blot. (J) Same as panels H and I except that endogenous Mbd3 polyubiquitylation was shown (*n* = 3). Values correspond to the average ± SD. Statistical *t* test analysis was performed to calculate significance (*p < 0.05, **p < 0.005, ***p < 0.0005; not significant (ns), *p* > 0.05). All quantification data underlying panels A, C, E, F, and G can be found in S2 Data.

To characterize Smek and Mbd3 expression during neural differentiation, we examined protein and mRNA levels after withdrawal of bFGF from NPC culture media. In NPCs, Mbd3 protein levels gradually decreased by day 1 of differentiation, while Mbd3 protein levels did not decrease in *Smek1* and *Smek2* single *KO* or *dKO* NPCs (Fig 3A–3C and S5A, S5B Fig). However, Mbd3 mRNA levels did not change during differentiation in both *WT* and *Smek1*, *Smek2*, or *Smek1/2 dKO* NPCs, suggesting that changes in Mbd3 protein levels that occur during differentiation require Smek (Fig 3D and 3E and S5A Fig, lower panel). These observations led us to further analyze Mbd3 stability. We found that Mbd3’s half-life was ~6 h in NPCs and 293T cells in which new protein synthesis was inhibited and significantly prolonged in the presence of the proteasomal inhibitor MG132 (Fig 3F). Endogenous Mbd3 protein levels in NPCs were increased by MG132 treatment (Fig 3G). Overexpressed Mbd3 protein was polyubiquitylated, and polyubiquitylated proteins accumulated in cells treated with MG132 or MG-101, respectively (Fig 3H and 3I and S5C, S5D Fig). Furthermore, endogenous Mbd3 in NPCs was found to be polyubiquitylated as well (Fig 3J).

**Fig 4:**
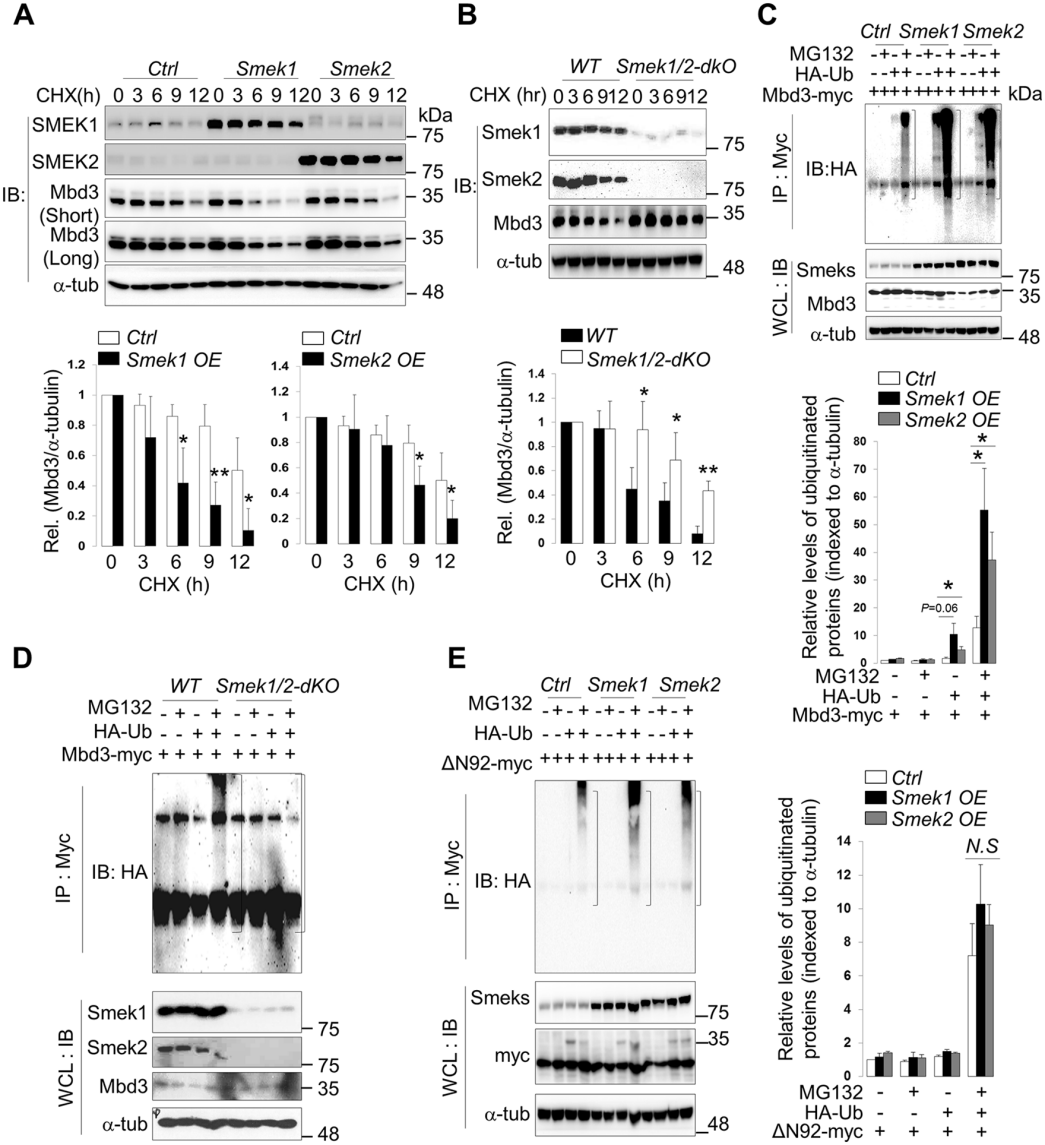
Suppressor of Mek null (Smek) promotes methyl-CpG–binding domain protein 3 (Mbd3) degradation and polyubiquitylation. (A) HEK293 cells and cell lines stably expressing Smek1 or Smek2 were treated with cycloheximide (CHX) for indicated times and whole cell lysates were prepared for immunoblotting (*n* = 4). (Lower) Quantification of band intensities is shown in the lower panel. (B) *Wild-type* and *Smek1/2 dKO* NPCs were treated with CHX for indicated times (h, hours) and harvested, and then lysates were immunoblotted (*n* = 3). (Lower) Quantification of band intensities. OE, overexpression; Ub, ubiquitin. (C) Ubiquitylation assay of Mbd3 using control and Smek1 or Smek2 stable HEK293T transfected with Mbd3-myc and HA-Ub expression vectors and treated 1 d later with MG132 for 6 h (upper blot) and IB for indicated proteins (lower blot). (Lower) Quantification of band intensities (*n* = 3). (D) Same as panel C except using *WT* or *Smek1/2 dKO* NPCs (*n* = 2). (E) Same as panel D except using cells transfected with Mbd3(ΔN92)-myc and HA-Ub expression vectors at control and Smek1 or Smek2 stable cell lines (*n* = 2). In (A–C and E), quantification of band intensity was done using ImageJ. Data are presented as average ± SD. Statistical *t* test analysis was performed (*p < 0.05, **p < 0.005, ***p < 0.005; not significant (ns), *p* > 0.05). All quantification data underlying panels A, B, C, and E can be found in S2 Data.

To determine whether Smek regulates Mbd3 protein stability, we monitored endogenous Mbd3 protein levels in *wild-type* NPCs, in HEK293T cell lines stably overexpressing Smek1 or Smek2 (S5E and S5F Fig), or in NPCs derived from *wild-type* and *Smek*1/2 *dKO* embryonic mouse brains (Fig 4A and 4B). Mbd3 protein turnover rate was increased by overexpression of either Smek1 or Smek2 and decreased upon Smek loss (Fig 4A and 4B). Consistent with Mbd3 degradation, Smek1 or Smek2 overexpression in HEK293T cells significantly promoted Mbd3 polyubiquitylation (Fig 4C and S6A Fig). Furthermore, Mbd3 was ubiquitylated in *wild-type* NPCs but not in *Smek1/2 dKO* NPCs (Fig 4D). To further assess the effects of Smek expression on Mbd3 degradation, we examined Mbd3 ubiquitylation following expression of the Mbd3 ΔN92 mutant, which cannot interact with Smek. Polyubiquitylation of this mutant was not significantly changed by overexpression of either Smek1 or Smek2 (Fig 4E and S6B Fig). These results suggest that interaction with Smek destabilizes Mbd3.

### Smek regulates enrichment of Mbd3/NuRD complex on neuronal gene loci and consequent gene expression

**Fig 5:**
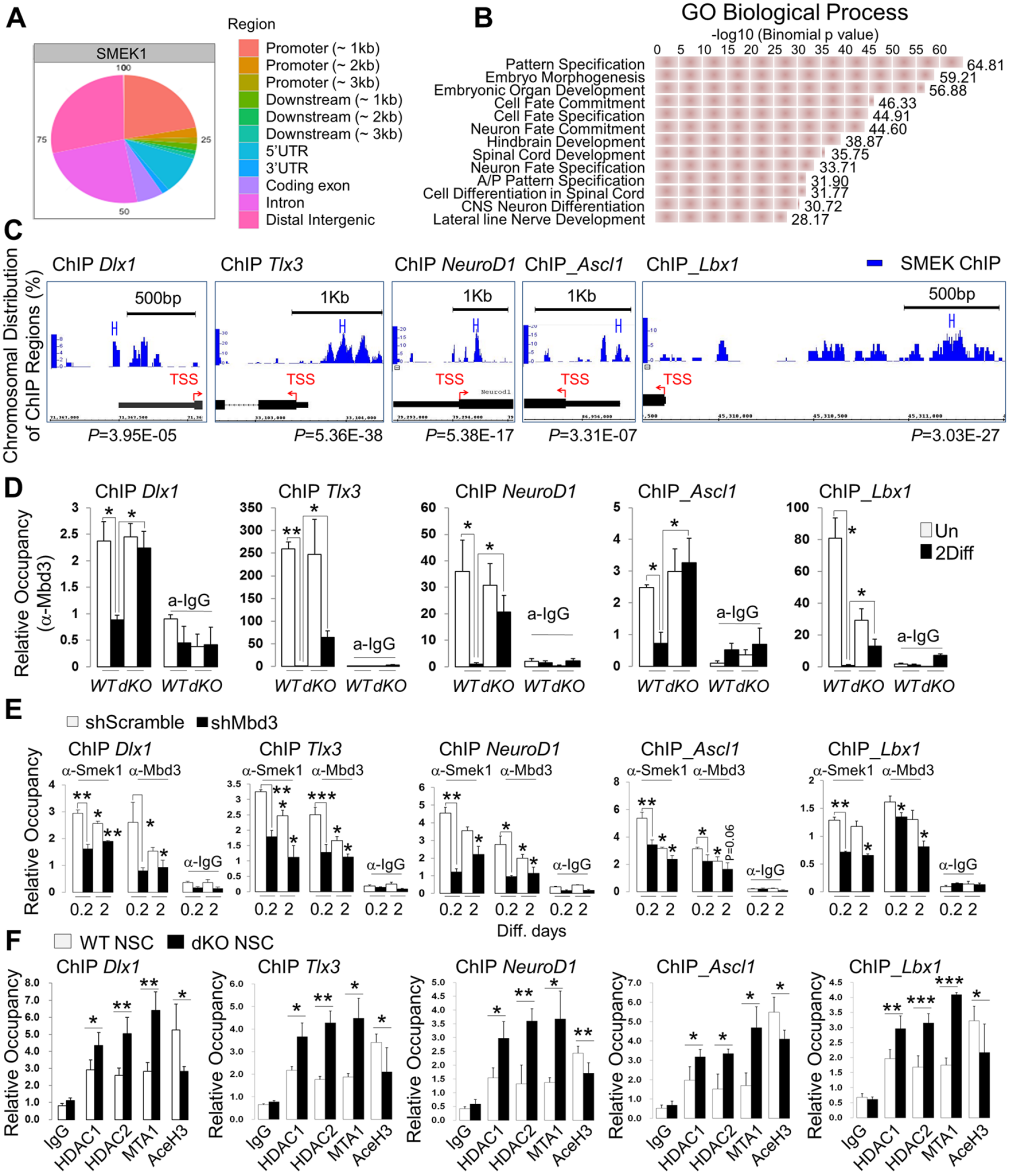
Suppressor of Mek null (Smek) and methyl-CpG–binding domain protein 3 (Mbd3) colocalize on neuronal gene promoters. (A) Smek1-chromatin immunoprecipitation sequencing (ChIP-seq) analysis in NPCs. Distribution of Smek1-binding peaks in NPCs. Proximal promoter regions are defined as sequences within 3 kb of the transcription starting site (TSS) of annotated genes. (B) Gene ontology (GO) analysis of annotated genes at Smek1-binding sites. (C) Smek1-binding peaks in NPCs in differentiation genes such as *Dlx1*, *Tlx3*, *NeuroD1*, *Ascl1*, and *Lbx1*. (D) ChIP-quantitative PCR (qPCR) analysis of Mbd3 occupancy at a Smek-binding locus in undifferentiated or differentiated conditions in *WT* (*n* = 3) and *Smek1/2 dKO* (*n* = 3) NPCs. (E) ChIP-qPCR analysis of Smek1 and Mbd3 occupancy at a Smek-binding locus in 0.2 or 2 d differentiated conditions in NPCs knocked down by shScramble (*n* = 3) and shMbd3 (*n* = 3) NPCs. (F) ChIP-qPCR analysis of HDAC1, HDAC2, MTA1, and acetyl histone H3 occupancy at a Smek-binding locus in undifferentiated or differentiated conditions in *WT* (*n* = 3) and *Smek1/2 dKO* (*n* = 3) NPCs. In panels D–F, immunoglobulin G (IgG) ChIP served as a negative control. Values are normalized to input control and represent average ± SD. *t* test analysis was performed to calculate the statistical significance (*p < 0.05, **p < 0.005). The ChIP-seq dataset for panels A–C can be found in S1 Data and all individual quantification data for panels D–F can be found in S2 Data.

Smek has a nuclear localization signal (NLS) and is nuclear localized [15], suggesting a potential role in regulating transcription. To determine whether the Smek protein is associated with chromatin—and if so, whether it is enriched on chromatin loci of neurogenesis-associated genes—we performed chromatin immunoprecipitation sequencing (ChIP-seq) in NPCs using a Smek1 antibody. Genome-wide binding profiling demonstrated that Smek proteins bind to chromatin loci of genes related to organ morphogenesis, cell-fate determination, and CNS development and differentiation (Fig 5A and 5B and S7A, S7B Fig). Furthermore, Smek specifically bound proximal promoter regions and gene bodies of neuronal genes such as *Dlx1*, *Dlx2*, *Tlx3*, *NeuroD1*, *Ascl1*, and *Lbx1*, which were known to highly express in neuron or proneuronal cells (Fig 5A–5C and S7C, S7D Fig). Unlike Smek, which lacks a known DNA-binding motif, Mbd3 exhibits the Mbd domain, which reportedly binds 5′-hydroxymethyl cytosine (5′-hmC) regions [20]. We therefore asked whether Smek and Mbd3 share similar genomic regions in NPCs, initially by determining whether Mbd3 binds neuronal gene promoters that Smek binds to. To do so, we undertook ChIP-qPCR with a Mbd3 antibody in *wild-type* and *Smek1/2 dKO* NPCs cultured in undifferentiation or differentiation conditions. This analysis confirmed enrichment of Mbd3 on the Smek-bound loci of genes including *Dlx1*, *Dlx2*, *Tlx3*, *NeuroD1*, *Ascl1*, and *Lbx1* in undifferentiated conditions (Fig 5D and S7C Fig). Moreover, Mbd3 enrichment on these gene loci significantly decreased under differentiation conditions in *wild-type* NPCs but was unchanged in *Smek1/2 dKO* NPCs under the same conditions (Fig 5D and S7C Fig). Then we asked whether enrichment of the Smek1 protein on chromatin loci of neurogenesis-associated genes is dependent on Mbd3 protein, and we performed ChIP-qPCR with Smek1 and Mbd3 antibodies in shScramble or shMbd3 knockdown (KD) NPCs cultured in undifferentiation or differentiation conditions. These results demonstrated that occupancy of Smek1 on the promoters of *Dlx1*, *Dlx1as*, *Tlx3*, *NeuroD1*, *Ascl1*, and *Lbx1* genes, but not *Gfap* genes, is dependent on Mbd3 protein (Fig 5E and S7F Fig). Mbd3 has been reported to represses transcription by recruiting the NuRD complex to target gene loci [20]. Co-IP experiments showed that Mbd3 interacted with MTA1, RbAP46, HDAC1, and HDAC2, which are components of NuRD complex (S7G Fig). We then undertook ChIP-qPCR with HDAC1, HDAC2, MTA1, and acetyl histone H3 antibodies using *wild-type* and *Smek1/2 dKO* NPCs cultured in differentiation conditions. ChIP analysis revealed that enrichments of NuRD components HDAC1, HDAC2, and MTA1 to target gene loci were significantly increased in *Smek1/2 dKO* NPCs when compared with those of the *wild type* (Fig 5F). Inversely, the amount of acetyl histone H3 was decreased (Fig 5F). These findings suggest that Smek inhibits enrichment of Mbd3/NuRD complex to neurogenesis-associated gene loci and increases acetyl histone H3 activity for their gene transcription during neurogenesis. These data also suggest that NuRD activity is dependent on Mbd3 ability, which can bind to target DNA, and Smek, as an upstream regulator of Mbd3/NuRD complex, promotes Mbd3 degradation, potentially allowing transcription of neuronal differentiation–associated genes by disrupting association and enrichment of Mbd3/NuRD complex on target gene loci.

### Mbd3 inhibits neuronal but not glial cell differentiation

**Fig 6:**
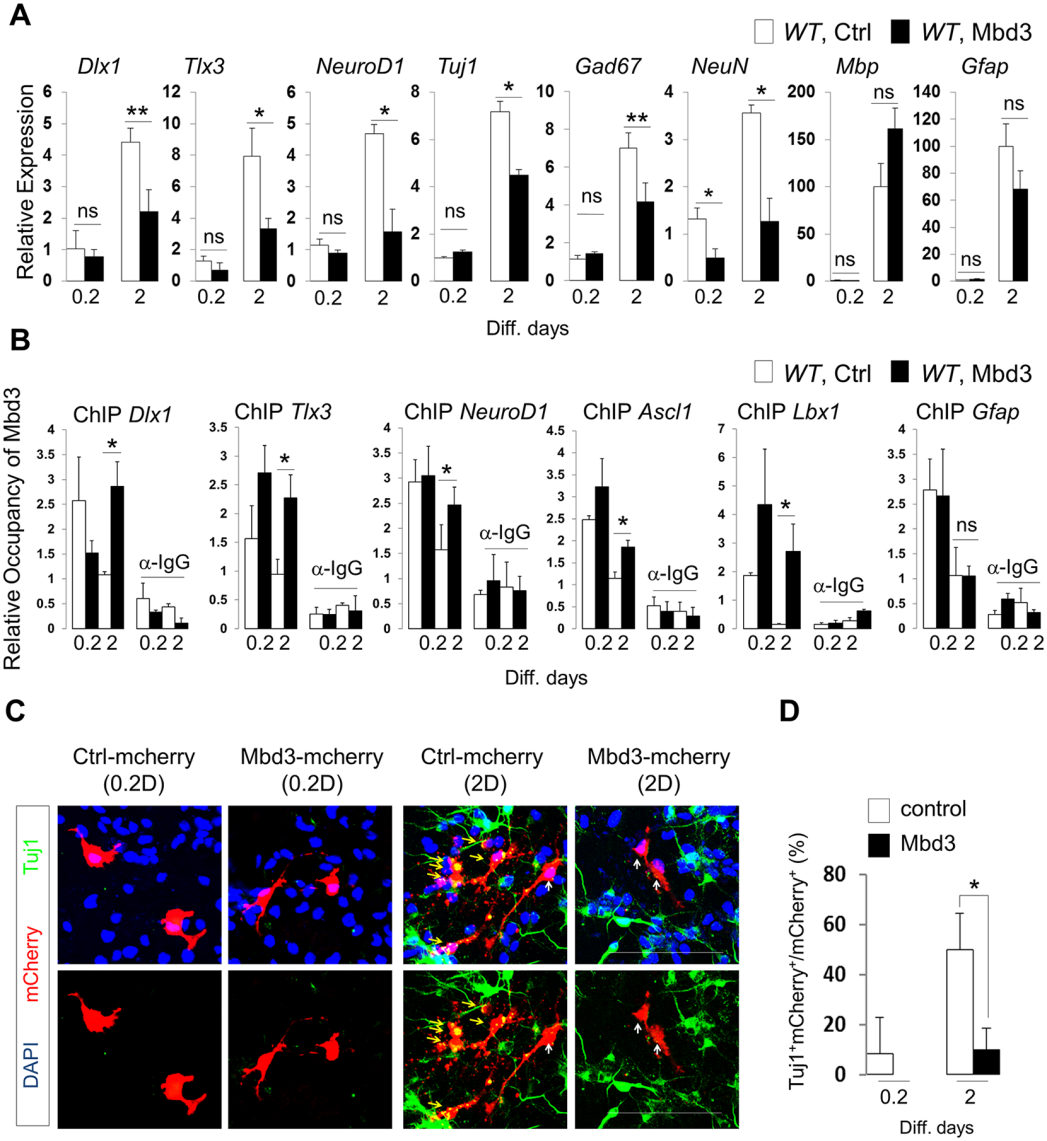
Effects of methyl-CpG–binding domain protein 3 (Mbd3) overexpression on neuronal gene expression and promoter occupancy during differentiation of neural progenitor cells (NPCs). (A) NPCs were electroporated with either pUltra-hot-control-mcherry or pUltra-hot-Mbd3-mcherry lentiviral vector and grown for 0.2 or 2 d in N2 medium with basic fibroblast growth factor (bFGF). Quantitative PCR (qPCR) analysis was used to measure the indicated transcript levels. (B) Mbd3 overexpression in *WT* NPCs increases Mbd3 occupancy of neuronal gene promoters but not that of *Gfap*, as determined by chromatin immunoprecipitation (ChIP)-qPCR. (C) Immunostaining to detect Tuj1 (green) and Mbd3-mcherry (red) expression. Nuclear staining is shown by 4',6-diamidino-2-phenylindole (DAPI) (blue). Scale bar, 100 μm. Yellow arrows indicate Tuj1/mcherry double-positive cells, and white arrows indicate Tuj1-positive cells. (D) Quantification of panel C. Data are presented as average ± SD. *t* test analysis was performed to calculate significance (*p < 0.05, ***p < 0.0005; not significant (ns), *p* > 0.05). All individual quantification data underlying panels A, B, and D can be found in S2 Data.

Our findings suggest that Mbd3 regulates expression of neuronal target genes in NPCs, an activity modulated by Smek. To assess whether Mbd3 represses neurogenesis-associated target genes, we overexpressed full-length or mutant (ΔN92) Mbd3 in NPCs and then induced differentiation over 2 d. Full-length Mbd3 (but not mutant form) overexpression attenuated *Dlx1*, *Tlx3*, *NeuroD1*, *Tuj1*, *Gad67*, and *NeuN* gene expression, all neuronal lineage markers, but had no effect on glial cell differentiation or gene expression (Fig 6A and S8A–S8D Fig). As noted above, Mbd3 bound specifically to the *Dlx1*, *Tlx3*, *NeuroD1*, *Ascl1*, and *Lbx1* gene loci, and this association decreased upon induction of differentiation conditions (Fig 5D). Thus, we asked whether decreased neuronal gene expression following Mbd3 overexpression paralleled increased occupancy of Mbd3 on target promoters (Fig 6B). As expected, the amount of overexpressed Mbd3 bound to gene promoters in NPCs in differentiation conditions over 2 d was similar to that seen in nondifferentiation conditions, leading to attenuate *Dlx1*, *Tlx3*, *NeuroD1*, *Tuj1*, *Gad67*, and *NeuN* gene expression, all markers of neuronal lineage (Fig 6A and S8B Fig). In contrast, there was little or no accumulation of Mbd3 on the *Gfap* promoter under the differentiation condition over 2 d for NPCs after Mbd3 overexpression, suggesting that Mbd3 blocks neuronal rather than glial cell differentiation (Fig 6B and S8B Fig). Immunocytochemistry analysis also showed that Mbd3 overexpression prevented NPC neuronal differentiation but did not affect astrocyte differentiation (Fig 6C, 6D and S8C, S8D Fig). Moreover, in both control and Mbd3-overexpressing NPCs, Nestin staining was comparable in staining intensity (S8C Fig). These data suggest that Mbd3 is a novel regulator for neuronal cell-fate determination of NPCs.

### Mbd3 loss rescues neurogenesis defects and promotes neuronal differentiation

**Fig 7:**
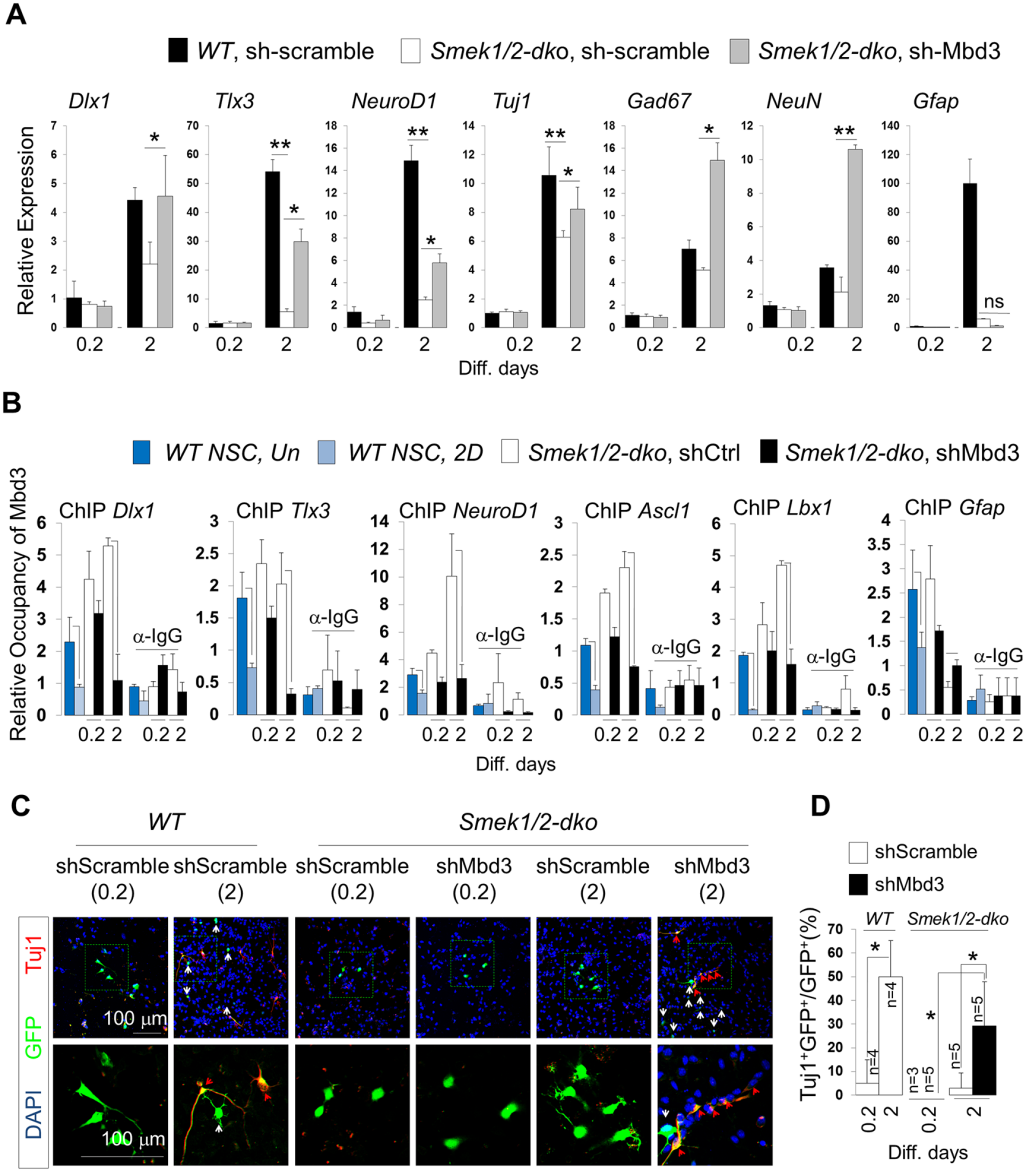
Effect of methyl-CpG–binding domain protein 3 (Mbd3) knockdown on neuronal gene expression and promoter occupancy over the course of differentiation of *Suppressor of Mek null double knockout* (*Smek dKO*) neural progenitor cells (NPCs). (A) *Wild-type (WT)* or *Smek1/2 dKO* NPCs were electroporated with either control pLKO3G-shScramble or pLKO3G-shMbd3 lentiviral vector and grown for 2 d in N2 medium with basic fibroblast growth factor (bFGF). qPCR analysis was performed to detect indicated mRNAs (*n* = 3 or 6). (B) Mbd3 knockdown in *Smek1/2 dKO* NPCs decreases Mbd3 occupancy of *Dlx1*, *Tlx3*, *NeuroD1*, *Ascl1*, and *Lbx1* promoters but not that of *Gfap*, as determined by chromatin immunoprecipitation-quantitative PCR (ChIP-qPCR) (*n* = 3). (C) Immunostaining to detect Tuj1 (red) and enhanced green fluorescent protein (EGFP) (green) expression. Nuclear staining is shown by 4',6-diamidino-2-phenylindole (DAPI) (blue). Scale bar, 100 μm. Red arrows indicate Tuj1/EGFP double-positive cells, and white arrows indicate EGFP-positive cells. (D) Quantification of panel C. All data are presented as average ± SD. *t* test analysis was performed to calculate significance (*p < 0.05, **p < 0.005; not significant (ns), *p* > 0.05). All individual quantification data underlying panels A, B, and D can be found in S2 Data.

**Fig 8:**
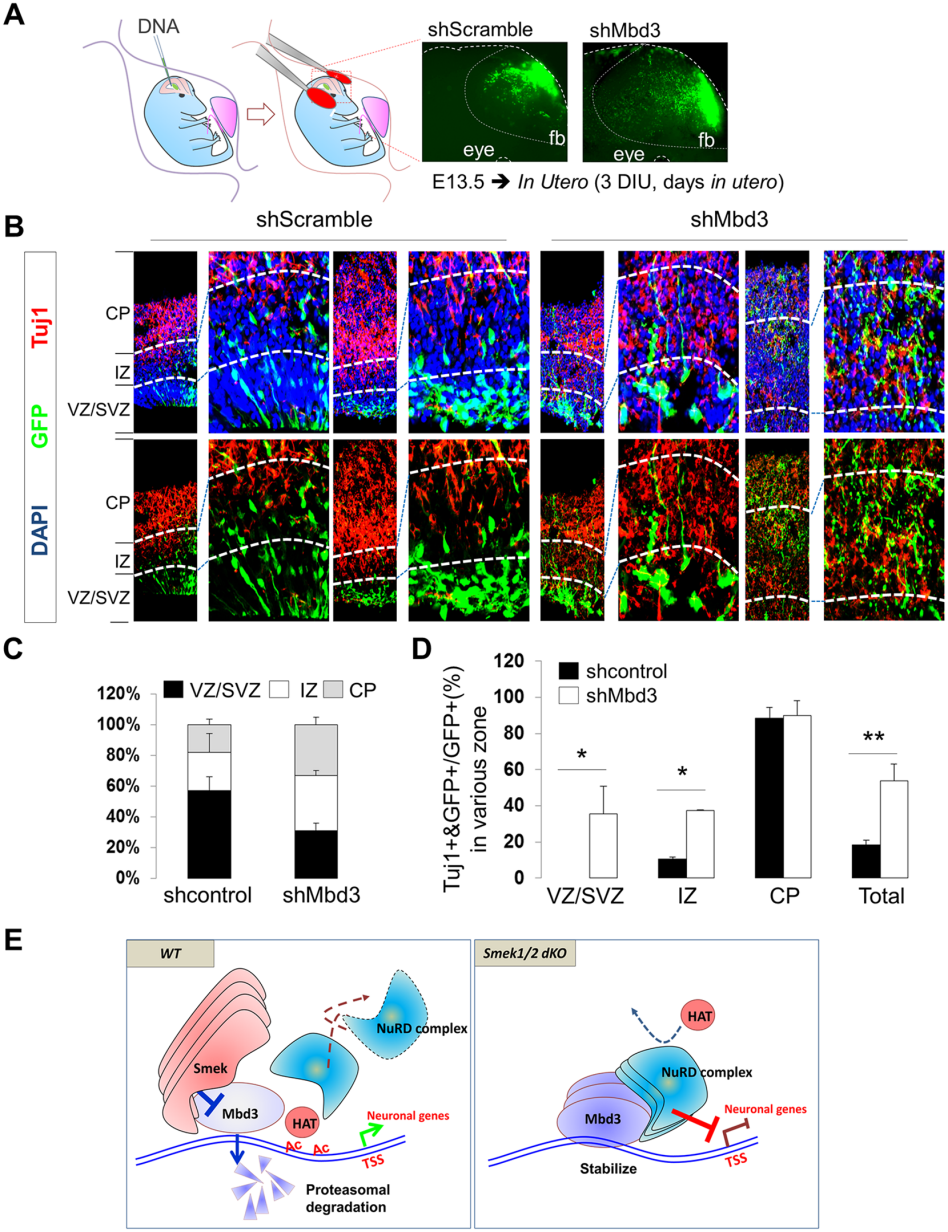
Effect of methyl-CpG–binding domain protein 3 (Mbd3) knockdown on the embryonic mouse cortex. (A) Schematic of injection site (green) and electrode position for in utero electroporation. Green fluorescent images indicates enhanced green fluorescent protein (EGFP)-labelled shScramble or shMbd3 expression in electroporated embryo brain (right panel). Fb, forebrain. (B) Confocal images showing immunofluorescent labeling of Tuj1 (red) and EGFP (green) in sections of the embryonic brain electroporated in utero. Nuclear staining is shown by 4',6-diamidino-2-phenylindole (DAPI) (blue). Scale bar, 50 μm. (C) Analysis of the percentage of migrating cells expressing EGFP in the brain’s ventricular zone (VZ)/subventricular zone (SVZ), intermediate zone (IZ), and cortical plate (CP) compartments in embryos electroporated either with control shScramble (*n* = 3) or shMbd3 (*n* = 4). (D) The effect of Mbd3 knockdown on neurogenesis was evaluated by counting Tuj1/EGFP double-positive cells versus total EGFP-positive cells in VZ/SVZ, IZ, and CP compartments. (E) A model on the role of the Smek-Mbd3-NuRD axis in cell-fate determination in cortical progenitor cells to neuronal cells during neurogenesis. Data are presented as average ± SD. Ac, acetylation; TSS, transcription starting site. *t* test analysis was performed to calculate statistical significance (*p < 0.05, **p < 0.005). All individual quantification data underlying panels C and D can be found in S2 Data.

To further investigate whether the Smek-Mbd3 axis regulates neurogenesis, we knocked down endogenous Mbd3 using an shMbd3 lentiviral vector in cultured *Smek1/2 dKO* NPCs and then induced differentiation for 2 d. Mbd3 KD significantly rescued effects of *Smek* loss on *Dlx1*, *Tlx3*, *NeuroD1*, *Tuj1*, *Gad67*, and *NeuN* expression but had no effect on astrocyte differentiation or gene expression (Fig 7A). Increased neuronal gene expression seen following Mbd3 KD was accompanied by decreased occupancy of target gene promoters by Mbd3 (Fig 7B). The epistatic relationship of Mbd3 and Smek in neurogenesis was analyzed by Mbd3 knockdown in *Smek1/2 dKO* NPCs. Mbd3 shRNA were expressed from the same vector that coexpressed enhanced green fluorescent protein (EGFP). The percentage of EGFP and Tuj1 double-positive cells among EGFP-positive cells was increased in cultures expressing Mbd3 shRNA but not in cells expressing Scramble shRNA (Fig 7C). We next assessed Mbd3 function in neurogenesis using an in utero electroporation system. Electroporated embryos were readily identifiable by EGFP expression (Fig 8A). About 74% of total EGFP-positive Mbd3 knockdown cells migrated toward the IZ or CP, while only ~39% of EGFP-positive control cells showed a similar migration pattern (Fig 8B and 8C). Quantitative analyses showed that the number of Tuj1-positive cells significantly increased in the VZ, SVZ, and IZ regions in Mbd3 KD EGFP-positive cells relative to control EGFP-positive cells (Fig 8D). These results strongly suggest that Mbd3 regulates NPC neuronal differentiation in the VZ or SVZ during cortical development.

## Discussion

Here, we have analyzed mouse embryos lacking functional *Smek1* and *Smek2* genes as well as cultured NPCs derived from those animals to understand *Smek* function during cortical development. We discovered that *Smek1/2 dKO* NPCs exhibit significantly reduced capacity for neuronal differentiation and increased self-renewal activity. Furthermore, we employed a Y2H screen to search for Smek binding partners and identified Mbd3 as a novel Smek-interacting protein (Fig 2A and S2 Table). Importantly, we observed that Smek promotes Mbd3 protein degradation and reduces Mbd3 occupancy of neural differentiation–associated gene promoters, likely increasing transcription of those genes via inhibiting recruitment of the repressive NuRD complex.

Interestingly, in the developing CNS, increased Mbd3 instability had an effect only on neuronal differentiation, with little or no effect on glial cell fate (Figs 6, 7 and S7F and S8B–S8D Figs). We could not determine the molecular mechanism by which the Smek-Mbd3 axis specifically regulates neuronal cell-fate determination but not glial cell fate. In our previous study, we found that protein phosphatase PP4c interacts with Smek and this complex suppressed Par3 activity for differentiation of NPCs [15]. PP4c is known to regulate neuronal cell-fate determination and organization of early cortical progenitors in the ventricular zone of the embryo brain by modulating spindle orientation during mitosis [24], and we could confirm the role of PP4c in neuronal differentiation of NPC by a PP4c loss-of-function study (S9A Fig). Interestingly, knockdown of PP4c significantly abolished neuronal cell as well as glial cell differentiation of NPCs, similar to Smek loss of function, and this finding suggests that Smek/PP4c/Par3 might have a different biological function from Smek/Mbd3 in at least regulating glial cell gene expression of NPCs (Fig 1E and S9A Fig). Moreover, Par3 regulation of Smek/PP4c during neurogenesis exclusively occurs in a cytosolic fraction but not in the nucleus of NPCs [15]. However, Smek and Mbd3 expression and transcriptional repression of Mbd3/NuRD complex mainly occurs in the nucleus of NPCs (Fig 2C and 2D). Our preliminary investigation of the relationship between Smek-PP4c complex and Mbd3 protein stability also reveals that loss of PP4c could not affect Smek-mediated Mbd3 polyubiquitylation (S9B Fig). In addition, ChIP-seq and ChIP-qPCR data show that Smek and Mbd3 are not significantly enriched at *Gfap* gene loci (S7F Fig). Thus, overall data suggest that the Smek-Mbd3 axis likely functions independently of the Smek-PP4c-Par3 axis, at least in regulation of *Gfap* gene expression, and that the Smek-Mbd3 interaction plays a crucial role in neuronal cell-fate determination in NPCs.

So far, five vertebrate MBD proteins have been identified as members of the MBD protein family: Mbd1, Mbd2, Mbd3, Mbd4, and MECP2 [25, 26], and these members are more highly expressed in the brain than in other tissues, leading investigators to hypothesize that they may play a critical role in normal brain development and in behavior [27, 28]. Our data indicate that Mbd3 represses neurogenesis and likely functions differently from other family members. For example, Mbd2 and Mbd3 are closely related and share a highly conserved methyl-CpG–binding domain, but mouse studies indicate that the two proteins are not functionally redundant [24], possibly because Mbd3 specifically recognizes methylated DNA, especially, 5′-hydroxymethylcytosine (5′-hmC) [19]. Deletion of Mbd3 gene in neural progenitor cells leads to generation of neurons expressing both deep- and upper-layer markers [23], suggesting that Mbd3 is required to maintain appropriate transcription in progenitor and neurons during neural development. A recent study suggests that Mbd3 may fine-tune expression of both active and silent genes [29]. Other studies suggest that conversion of 5′-mC to 5′-hmC coincides with increased transcriptional activity by excluding Mbd proteins from target genes [30]. Consistent with these findings, we found that Mbd3 is specifically bound to neuronal gene loci, and our findings suggest that it is likely released from these loci by Smek during NPC differentiation (Fig 5D).

Mbd3 is a subunit of the NuRD complex, which has nucleosome remodeling and histone deacetylase activities [17,18] and thus regulates gene expression. The molecular function of this complex has been extensively studied in the context of tumorigenesis, stem cell pluripotency, and brain development [23, 31–34]. Mbd3 mutation or abnormal expression may function in tumorigenesis by perturbing gene expression. Like Mbd3, other Mbd proteins, especially Mbd2 and Mbd4, are associated with progression of cancer such as colorectal cancer, albeit by different mechanisms [34–36]. Furthermore, Mbd3 knockdown during somatic cell reprogramming significantly increases reprogramming efficiency [31–33]. Although Mbd3 activity is likely relevant to pathologies seen in cancer, neurological disease, and developmental defects, mechanisms underlying its regulation remain unclear. We propose a novel function in which Mbd3 protein levels, depending on Smek activity, decrease during neurogenesis (Figs 2D, 3A–3C and S4C and S4D, S5A and S5B Figs). *Smek1/2 dKO* NPCs or the embryonic cortex show aberrantly high Mbd3 levels that may repress neuronal gene expression and underlie developmental defects seen in the latter. In accordance, we report that Smek promotes ubiquitylation and degradation of Mbd3 (Figs 3A–3C, 4A–4D and S5A–S5B, S6A Figs). Our data also indicate that Smek regulation of Mbd3 is not transcriptional, based on the lack of significant change in Mbd3 mRNA levels over NPC differentiation. Conversely, Mbd3 protein levels decreased during neuronal differentiation in the embryonic cortex starting at E12.5 in mice. In addition, decreased Mbd3 levels seen in cultured NPCs are blocked by MG132 treatment concomitant with accumulation of polyubiquitylated Mbd3. These results overall indicate that Mbd3 activity is regulated at the level of protein stability and that Smek likely governs this process. Changes in protein stability often constitute a more rapid means of regulating protein activity than does modulation of transcription. Therefore, regulation of Mbd3 protein stability might function epigenetically to recruit the NuRD complex to 5′-hmC–modified gene promoters. To our knowledge, this is the first report of regulation of an Mbd family protein by stability changes.

We also examined potential factors or complexes that might function in Smek-dependent Mbd3 degradation. To do so, we sought potential E3 ligase proteins that might catalyze Mbd3 ubiquitylation by using Biograph software and identified the E3 ligase TRIpartite Motif protein 33 (TRIM33) protein, which has an *N*-terminal Really Interesting New Gene (RING)-domain (S10 Fig). TRIM33 specifically targets phosphorylated nuclear proteins for degradation [37]. Interestingly, we have previously identified protein kinase C (PKC) lambda/iota (λ/ι), a serine/threonine kinase, as a binding partner of Smek1 from a mass spectrometry analysis [15]. PKC isoforms contain an NLS and contribute diverse cellular physiology [38–40]. Smek1/2 also have NLS sequences and are localized exclusively in the nucleus in interphase [15]. To further investigate the involvement of PKCλ/ι in the molecular mechanism for Mbd3 protein stability, we performed prediction of putative kinases for phosphorylation of Mbd3 protein by GPS (group-based prediction system) software 3.0 (http://gps.biocuckoo.org/). (S3 Table). Interestingly, we predicted PKCλ/ι as putative kinases for Mbd3 phosphorylation. Although it still remains unclear how Smek1/2 promotes ubiquitylation and stability of Mbd3, accumulating data and predictions suggest that nuclear-localized Smek1/2-PKCλ/ι complex with TRIM33 may function in Mbd3 ubiquitylation and degradation. Alternatively, Aurora-A protein, a serine/threonine kinase, reportedly physically associates with Mbd3 at centrosomes in early M phase in vivo and phosphorylates Mbd3 protein in vitro [41]. These findings suggest that Aurora-A may also be involved in the regulation of Mbd3 protein stability as a different mechanism from Smek-PKCλ/ι complex. This topic will be addressed in future studies.

Smek orthologues in *Drosophila* play a critical role in neuroblast mitosis [16]. In Smek-deficit neuroblasts, cell-fate determinants, such as Prospero and Miranda, are no longer localized to the cell cortex; instead, they are distributed in the cytoplasm of dividing neuroblasts [16]. As a result, asymmetric cell division and neurogenesis are defective. In the *Drosophila* system, another class of asymmetric cell division regulators are epigenetic modulators. However, it is not clear if Smek functions through epigenetic modulators. Our studies suggest that Smek and Mbd3 have the opposite function in the NPCs’ differentiation in vertebrate systems. Although these studies do not address asymmetric cell division, our research may shed light on asymmetric cell division and neurogenesis in *Drosophila* and mammals.

Our findings also highlight the importance of Smek/Mbd3 interaction in regulating NPC differentiation. Other studies suggest that Smek and Mbd3 may have overlapping functions or activities in brain development, stem cell activity, and regulation of transcription [15,16,20,21,42]. Our studies support a functional relationship of Smek and Mbd3 in NPCs. Our mapping analysis shows that Smek/Mbd3 interaction is mediated by the Mbd domain of Mbd3. Immunofluorescence analysis confirmed close proximity of these proteins in the NPC nucleus, and we have observed coincident expression of Smek and Mbd3 in the mouse embryonic brain [16,21]. Finally, we found that Smek and Mbd3 target the same neuronal gene loci for regulating transcription (Fig 5D). Further analysis suggests a model in which Smek regulates target gene transcription by regulating Mbd3 protein stability, interaction with NuRD components, and recruitment of Mbd3/NuRD complex to the promoters of target genes. *Smek1/2 dKO* exhibits reduced neuronal differentiation and decreased expression of *Dlx1*, *Dlx2*, *NeuroD1*, *Tuj1*, *Gad67*, *NeuN*, and stabilizing Mbd3 protein, while Mbd3 overexpression attenuated Smek-mediated neuronal differentiation (Figs 1E, 3A–3C, 6A, 6C and 6D). Thus, this study is significant not only for demonstrating Smek-mediated Mbd3 protein degradation but also in providing evidence that Smek/Mbd3 interaction regulates neuronal gene expression and neuronal differentiation during cortical development. In conclusion, we report functional interaction of Smek with Mbd3 in neuronal differentiation of NPCs.

## Materials and methods

### Ethics statement

All animal procedures were approved by the Institutional Animal Care and Use Committee (IACUC) and the National Institutes of Health (IACUC protocol number: 11489). Mouse embryos and primary neural progenitor cells were obtained from a deceased pregnant mouse following CO2 asphyxiation. For in utero electroporation experiments, timed-mated pregnant mice had been anesthetized with Avertin (2.5%) (Sigma, St. Louis, MO) following IACUC instruction. At the experimental endpoints, mice were euthanized by CO2 asphyxiation.

### Animals

*Smek1/2 dKO* mice were generated using gene trap mutant ES cells obtained from the Gene Trapping Consortium. Gene trap vectors were targeted between exons 3 and 4 of the *Smek1* gene and between exons 10 and 11 of the *Smek2* gene, respectively. *Smek1* and *Smek2* mutant ES cells (E14) were injected into mouse blastocysts and chimeric mice were backcrossed with C57BL/6 mice. *Smek1/2 dKO* mice were generated by crossing C57BL6J-*Smek1*^*+/-*^ with *Smek2*^*+/-*^ mice. After six generations, mice were used for analysis. Although *Smek1/2 dKO* mice can die at later stages of embryonic development, we were able to obtain *dKO* embryos as late as E14.5 with a normal Mendelian distribution. Thus, we have conducted functional analysis of *Smek1/2 dKO* embryos at E11.5, E12.5, and E14.5. Embryos and pups of *wild-type* and heterozygous *KO* mice were collected from timed-mated pregnant females.

### Antibodies and reagents

Antibodies used in this study were anti-Smek1 (rabbit polyclonal 1:500 dilution) anti-Smek2 (rabbit polyclonal 1:500 dilution), anti-Flag (mouse monoclonal 1:2,000 dilution) (Sigma), anti-Mbd3 (rabbit polyclonal 1:500 dilution), anti-CDH3 (rabbit polyclonal 1:500 dilution), anti-RbAP46 (rabbit polyclonal 1:2,000), anti-GFAP (rabbit polyclonal 1:200 dilution), anti-MTA1 (rabbit polyclonal 1:2,000 dilution), anti-RbAp46 (rabbit polyclonal 1:2,000 dilution), anti-HDAC1/HDAC2 (mouse monoclonal 1:2,000 dilution) (Cell Signaling Technology, Beverly, MA), anti-HA (rabbit polyclonal 1:500 dilution), anti-GST (rabbit polyclonal 1:500 dilution), anti–α-tubulin (mouse monoclonal 1:5,000 dilution), anti-HA (mouse monoclonal 1:3,000 dilution) (Santa Cruz Biotechnology, Santa Cruz, CA), anti-MAP2ab (rabbit polyclonal 1:200 dilution) (Chemicon, Temecula, CA), anti-NeuN (rabbit polyclonal 1:200 dilution) (EMD Millipore, Billerica MA), anti-Nestin (mouse monoclonal 1:350 dilution) (BD Biosciences, San Jose, CA), anti-Tuj1 (mouse polyclonal 1:200 dilution) (Covance, Princeton, NJ), anti-Tbr1 (rabbit polyclonal 1:200 dilution), and anti-Pax6 (rabbit polyclonal 1:200 dilution) (Abcam Ltd, Cambridge, MA). Secondary antibodies were anti-rabbit Alexa Fluor 488-, anti-mouse Alexa Fluor 488-, anti-rabbit Alexa Fluor 555-, or anti-mouse Alexa Fluor 555-conjugated IgG (1:200 dilution) (Molecular Probes, Eugene, OR). bFGF was purchased from PeproTech (Rocky Hill, NJ). The protease inhibitor cocktail was from Roche Applied Science (Indianapolis, IN). To HDAC inhibition, Trichostatin A (TSA) and 4-(dimethylamino)-N-[6-(hydroxyamino)-6-oxohexyl]-benzamide (DHOB) were purchased from Santa Cruz Biotechnology. TRIzol, Protein A/G agarose beads, and DAPI were from Sigma. The ECL Kit and KOD Hot Start DNA polymerase were from EMD Millipore. Glutathione magnetic beads, phenol:chloroform:isoamyl alcohol, and the First Strand cDNA Synthesis Kit were from Thermo Fisher Scientific (Rockford, IL).

### Immunoprecipitation and western blotting

Cells were gently lysed with IP buffer (50 mM Tris-HCl, pH 7.4, 130 mM NaCl, 10mM NaF, 2 mM EGTA, 2 mM EDTA, 0.5% Triton X-100, 0.5% NP-40, 5% glycerol, 1 mM dithiothreitol [DTT], and a protease inhibitor cocktail) for 1 h on ice and then centrifuged at 14,000 rpm at 4°C for 15 min. The supernatant was collected and precleared with 30 μl of Protein A/G beads (Santa Cruz Biotechnology) for 2 h, and then precleared lysates were incubated with 4 μg of each specific antibody overnight at 4°C. Lysates were then incubated with 30 μl of Protein A/G beads for 4 h at 4°C. After immune complexes were washed six times with IP buffer, they were eluted by boiling for 3 min at 95°C in SDS sample buffer and separated on 10% SDS-PAGE. After blocking, membranes were incubated with primary antibody and then with a peroxidase-conjugated secondary antibody. Bound secondary antibody (anti-mouse or anti-rabbit 1:10,000) (Santa Cruz Biotechnology) was detected using the enhanced chemiluminescence (ECL) reagent (Santa Cruz Biotechnology).

### Immunohistochemistry and immunocytochemistry

For immunohistochemistry, embryonic brains were dissected and fixed in 4% parafomaldehyde (PFA) at 4°C, cryoprotected in 30% sucrose, embedded and frozen in Tissue Tek OCT compound, and sectioned at 30 μm on a cryostat. Sections were incubated with primary antibody at 4°C for 18 h. For immunocytochemistry, cells cultured on coverslips were fixed with 4% PFA/PBS for 30 min and immunostained after permeabilizing with 0.2% Triton X-100. Tissues and cells were incubated with secondary antibodies at room temperature for 1 h and counterstained in 4'-6-diamidino-2-phenylindole (DAPI) (Boehringer Mannheim, Mannheim, Germany), and images were visualized using confocal microscopy (LSM5 PASCAL; Zeiss, Jena, Germany). Values obtained from at least three independent experiments were averaged and reported as means ± SD. The two-tailed Student’s *t* test was used to compare two experimental groups.

### GST pull-down assay

DH5 bacteria were transformed with GST-tagged plasmids (*Mbd3*, *ΔN36*, *ΔN92*, *ΔC249*, *ΔC221*, *ΔC174*, and *ΔC93*) and protein expression was induced by addition of 0.5 mM isopropyl 1-thio-β-D-galactopyranoside (IPTG) at 25°C at mid-log phase. Cells were lysed with B-PER Bacterial Protein Extraction Reagent (Thermo Fisher Scientific), lysates were purified, and proteins were captured using with Glutathione magnetic beads. HEK293T cells were transfected with Flag-tagged Smek2 plasmid and lysed with lysis buffer for 1 h on ice. Cell lysates were centrifuged at 14,000 rpm at 4°C for 15 min, and collected supernatants were incubated with Glutathione magnetic beads bound to GST or GST proteins. Bound proteins were eluted by boiling for 3 min at 95°C in SDS sample buffer, followed by immunoblotting.

### Preparation and culture of neural progenitor cells

NPCs were prepared from E11.5 cortex of *Wild-type*, *Smek1*^*-/-*^, *Smek2*^*-/-*^, and *Smek1/2 dKO* mice in Hank’s balanced salt solution (HBSS) (Invitrogen) and cultured as described [43]. To maintain stem cell characteristics, NPCs were cultured in N2 medium containing bFGF for 4 d. Stemness of cultured NPCs was confirmed by Nestin and Sox2 expression. To induce NPC differentiation, cells were seeded and further cultured in the absence of bFGF2.

### Plasmids and shRNA transfection

NPCs derived from *Wild-type* or *Smek1/2 dKO* E11.5 forebrain were transfected with 4 μg pUltra-hot-Mbd3-flag or a Myc-tag vector for Mbd3 gain-of-function experiments and with pLKO3G-shMbd3 for Mbd3 loss-of-function experiments using Lipofectamine LTX and Plus Reagent (Invitrogen) or electroporation with an AMAXA nucleofector (Ronza AG, Basel, Switzerland). pLKO3G-shcontrol vector was used for negative control of pLKO3G-shMbd3 vector. After 12 h, transfection efficiency was confirmed to be >90% by monitoring mcherry or EGFP signaling. After two more days in differentiation conditions, cells were analyzed by qPCR or ChIP.

### Plasmids and shRNA

Mbd3 expression vectors were constructed by subcloning full-length mouse Mbd3 from a lentiviral FUIGW-Mbd3-Flag vector we previously created into XbaI/EcoRI sites of pUltra-hot (Addgene plasmid # 24130). For Mbd3-myc, the reverse primer included the full Myc sequence. PCR was carried out using the KOD Hot Start Polymerase Kit (EMD Millipore) with corresponding primer pairs. PCR products were ligated into double-digested pUltra-Hot vector and inserted ligations were confirmed by PCR and DNA sequencing (Genewiz, Inc). For the shRNA vector, the Mbd3 target sequence was designed based on the RNAi Consortium library top hits for mouse Mbd3. Details for pLKO3G shMbd3 and shcontrol construction are listed in S4 Table.

### Quantitative RT-PCR

Cells were harvested and total RNA was isolated using TRIzol reagent (Invitrogen). The SuperScript III qRT-PCR kit (Invitrogen) was used to synthesize cDNA from total RNA. Quantitative PCR was carried out using the ABI PRISM 7900 Sequence Detection System with SYBR Green Master Mix (iTaq) with conditions of 95°C for 10 min followed by 50 cycles at 95°C for 15 sec and 60°C for 3 sec. Samples were run in triplicate and *Dlx1*, *Dlx2*, *Tlx3*, *NeuroD1*, *Tuj1*, *Gad67*, *NeuN*, *Mbp*, *Gfap*, *Ascl1*, and *Id1* transcript quantitation was undertaken by comparing Cycle Threshold (Ct) values for each reaction with the *Gapdh* reference. Primer sets for quantitative PCR are listed in S5 Table.

### ChIP, ChIP-seq analysis

For the ChIP assay, NPCs derived from *wild-type* or *Smek1/2 dKO* E11.5 forebrain or transfected with 4 μg pUltra-hot-Mbd3-flag or a pUltra-hot-empty vector for Mbd3 gain-of-function experiments and with pLKO3G-shMbd3 or pLKO3G-shScramble for Mbd3 loss-of-function were treated with 1% formaldehyde for 10 min at room temperature and quenched with 0.125 M glycine for ten more minutes at room temperature. Cross-linked chromatin was sonicated to fragment DNA to 200–1,000 base pairs, and then immunoprecipitation was performed with rabbit anti-IgG, anti-Smek1 (Sigma), anti-Mbd3 (Cell Signaling), anti-HDAC1, anti-HDAC2, anti-MTA1, and acetyl histone H3 (Santa Cruz Biotechnology) antibodies overnight at 4°C, followed by incubation with 50 μl of magnetic Protein A/G Dynabeads (EMD Millipore). Abundance of sequences in immunoprecipitates was determined by PCR and normalized as a fold-value relative to input chromatin. Smek ChIP-seq data were analyzed with the MACS online tool, and *cis*-regulatory sequences were analyzed using the Genomic Regions Enrichment of Annotations Tool (GREAT) interface (http://bejerano.stanford.edu/great/public/html/). We also utilized the Intergrative Genomics Viewer (IGV v2.3) to visualize distribution of ChIP-seq–identified peaks in different genomic regions. Primer sets for ChIP-qPCR are listed in S6 Table.

### In utero electroporation

All procedures followed guidelines of the Institutional Animal Care and Use Committee (IACUC) and the National Institutes of Health. A total of 2.5% (w/v) avertin (1 g/ml solution of 2, 2, 2-Tribromoethanol, 97% in tert-amylalcohol [99%]; Aldrich, catalog numbers T4,840–2 and 24,048–6, respectively) in 0.9% saline was injected i.p. (15 μl/g of body weight) to anesthetize pregnant mice (E13.5). A laparotomy was performed, and the uterus with embryos was exposed. A total of ~2–5 μl of plasmid DNA (approximately 2 μg/ μl, dissolved in water) was injected into the lateral ventricle using a fine-glass microcapillary and a PV830 pneumatic PicoPump. Electroporation was performed using a Nepagene CUY21SC electroporator (amplitude, 50 V [E13.5]; duration, 50 ms; intervals, 150 ms). To deliver electrical pulses, tweezer-type circular electrodes (7-mm diameter) were used with the positive side directed to the medial wall of the ventricle into which DNA was injected. Uterine horns were repositioned in the abdominal cavity, and the abdominal wall and skin were sewed with surgical sutures. Mice were kept on a warm plate (37°C) for recovery. Two to three days later, embryos were taken from mothers and fixed with 4% (w/v) PFA (Sigma) in PBS (pH 7.4). After a 24 h fixation at 4°C, embryo brains were transferred to a 30% (w/v) sucrose solution in 4% PFA. Tissues were sectioned at 30 μm using a cryotome (Leica) and analyzed by immunohistochemistry.

### Yeast two-hybrid screening

*Smek2* DNA was fused in frame to the LexA bait vectors pBTM116 for use as bait in the yeast two-hybrid screen. Preys were expressed as fusions to the activation domain of GAL4 in pACT2 (BD Biosciences Clontech, Palo Alto, CA, US). Transformed bait strains with or without transformed prey strains were mated and analyzed by using β-galactosidase activity. The following preys were used: mbd3. The Saccharomyces cerevisiae strain L40 (MATa trp1 leu2 his3 LYS2:: lexA-HIS3 URA3::lexA-lacZ) was cotransformed with bait and prey plasmids using the PEI method and selected for histidine prototrophy on minimal medium, containing 2% glucose; 6.7% yeast nitrogen base (BD Diagnostic Systems, Sparks, MD, US); complete amino acid mixture lacking histidine, leucine, and tryptophan (Qbiogene, Carlsbad, CA, US); and 2% bacto agar (BD Biosciences, Franklin Lakes, NJ, US). Yeast transformants were grown for 3 d at 30°C.

### Statistical analysis

Statistical differences among groups were analyzed using Student’s *t* test and are indicated in each Fig as follows: *p < .05, **p < .005, and ***p < .0005. *p < .05 was considered statistically significant.

## Supporting information

S1 Fig Cortical development in *Smek1/2* double knockout (*dKO*) mice. (A, B) Gene trapped mutant *Smek1* and *Smek2* ES cell (E14) were injected into mouse blastocyst and chimeric mice were backcrossed with C57BL/6 mice. After 6 generation, mice were used for analysis. (A) cDNA was prepared from total RNA harvested from *wild-type* and *Smek1/2 dko* NPCs and expression of indicated genes was measured by RT-PCR. (B) Respective lysates were immunoblotted with indicated antibodies (n = 2). Quantification of band intensity was done using Image J. (C) Representative images showing MAP2 staining at E12.5 (see Fig 1A, right panel). (D-F) Coronal sections from E12.5 mouse brain stained with antibodies against Ki67 (red), Nestin (green), and MAP2 (red). Nuclei were counterstained with DAPI (blue). Scale bar: 100 mm. (E) Identification of self-renewal and proliferating NPCs by Ki67 and Nestin staining in E12.5 mouse cortex (*WT*, n = 7; *Smek1/2 dKO*, n = 5). (F) Quantification of S1E Fig. Data is quantified using the Image J software. Bar graphs represent means ± S.D. *P < 0.05 (unpaired Student *t*-test). Scale bar: 100 mm. The underlying data for panels B and F can be found in the S2 Data file.
(TIF)

S2 Fig Effects of *Smek1 KO* on mouse cortical development. (A) Coronal sections from *WT* or *Smek1 KO* cortex at E12.5 or E14.5 were stained with Tuj1 (red) and Tbr1 (green) antibodies. Nuclei were counterstained with DAPI (blue). Scale bar: 100 mm. (B) Quantification of staining for Tuj1+, Tbr1+, or double-positive cells using the Image J software. Bar graphs represent means ± S.D. (n = 3). *P < 0.05 (Student’s *t*-test). The underlying data for panel B can be found in the S2 Data file.
(TIF)

S3 Fig Effects of *Smek* loss on differentiation capacity of neural progenitor cells (NPCs). (A and B) *Smek1 KO*, *Smek1/2 dKO* or *Wild-type* NPCs were grown in N2 medium without bFGF for indicated days. cDNA was prepared from total RNA harvested from *WT*, *Smek1KO* and *Smek1/2 dKO* NPCs and expression of indicated genes was measured by RT-PCR (n = 2). Diff. (d), days in differentiation.
(TIF)

S4 Fig Smek interact with Mbd3 in vitro and in vivo. (A) Immunostaining with Mbd3 (red) and Smek2 (green) antibodies in HEK293 cells. DAPI (blue). Scale bars, 50 mm. (B) Immunoprecipitation (IP) using Flag or HA antibodies from lysates with either Flag-Smek1 or -Smek2 in the presence or absence of HA-Mbd3, or HA-Mbd3 plus control vector or Flag-Smek2 (n = 2). (C-D) Paraformaldehyde (PFA)-fixed, cyro-embedded coronal sections from E12.5 and E14.5 mouse cortex were stained with antibodies against Mbd3 (red), Smek1 (green or red) and Ki67 (green). Nuclei were counterstained with DAPI (blue). Yellow arrows indicate perinuclear localization of Smek1 in ventricular zone progenitor cells. Images were captured using a Zeiss confocal microscope. Scale bar: 25 or 100 mm. (D) Quantification of endogenous Mbd3 (green line) and Smek1 (red line) expression pattern was shown using the ZEN lite image software (http://www.zeiss.com/).
(TIF)

S5 Fig *Smek1/2 dKO* inhibits Mbd3 protein degradation. (A, upper panel) NPCs were grown in N2 medium without bFGF for indicated days, and lysates were immunoblotted with indicated antibodies (n = 2). (A, lower panel) cDNA was prepared from total RNA from *Wild-type* or *Smek1/2 dKO* NPCs, and indicated transcript levels were measured by RT-PCR (n = 2). (B) Paraformaldehyde (PFA)-fixed, cyro-embedded coronal sections from *Wild-type* or *Smek1/2 dKO* E12.5 mouse cortex were stained with antibodies against Mbd3 (red). Nuclei were counterstained with DAPI (blue). Images were captured using a Zeiss confocal microscope. Scale bars: 100 mm. (C) HEK293 cells were transfected with plasmids expressing Mbd3-Flag and HA-Ub, or Mbd3-Flag alone. At 24 hours after transfection, cells were treated with MG101 (25 μg/ml) for 12 hours before harvest. Lysates were prepared and immunoprecipitated using anti-Flag beads Mbd3 ubiquitylation was detected by immunoblotting with anti-HA antibody. Lysates were analyzed by immunoblotting for indicated proteins (n = 2). Ub, Ubiquitin. (D) Same as S5C Fig except using A/G beads incubated with anti-Mbd3 (n = 1). (E and F) HEK293 cells were infected with supernatants of lentivirus expressing *Smek1 or Smek2*. These cells were further selected during 2 weeks in medium contained puromycin (1ug/ml) and their expression was confirmed by immunoblotting with anti-Smek1 or anti-Smek2 antibodies (n = 2).
(TIF)

S6 Fig Effects of Smek1 overexpression on Mbd3 polyubiquitylation. (A) HEK293 cells and lines stably overexpressing *Smek1* were transfected with vector, Mbd3-Flag, and HA-Ub expression plasmids. One day later, cells were treated with MG132 for 6 hours, and lysates were immunoprecipitated with anti-myc beads (n = 4). (B) HEK293 cells and lines stably overexpressing *Smek1* were transfected with indicated constructs, treated with MG132 for 6 hours, and immunoprecipitated with myc-conjugated beads. Mbd3 ubiquitylation was detected by immunoblot with anti-HA antibody. Smek1, Mbd3, and a-tubulin in lysates were detected by immunoblotting (n = 2).
(TIF)

S7 Fig Function of annotated genes occupied by Smek1 based on ChIP-seq analysis. (A) Molecular function based on Gene ontology (GO) analysis. (B) Cellular function based on Gene ontology (GO) analysis. (C) (upper panel) Smek1 binding peaks in NPCs in differentiation genes such as *Dlx2*. Scale bar, 200 bp. (Lower panel) ChIP-qPCR analysis of Mbd3 occupancy at a Smek binding loci (*Dlx2* gene promoter region) in undifferentiated or differentiated conditions in *WT* (n = 3) and *Smek1/2 dKO* (n = 3) NPCs. IgG ChIP served as a negative controls. (D) Smek1 binding peaks in NPCs in differentiation genes such as *Dlx1*, *Tlx3*, *NeuroD1*, *Ascl1*, and *Lbx1*. (E) NPCs were transfected with pLKO3G-shMbd3 #1, #2, and #3, and one day later, Mbd3 and a-tubulin in lysates were detected by immunoblotting (n = 2). (F) ChIP-qPCR analysis of Smek1 and Mbd3 occupancy at a Smek binding locus (*Gfap* gene promoter) in undifferentiated or differentiated conditions in NPCs knock-downed by shscramble (n = 3) and shMbd3 (n = 3) NPCs. IgG ChIP served as a negative control. Values are normalized to input control and represent average ±SD. *t*-test analysis was performed to calculate the statistical significance (*P < 0.05, **P < 0.005). (G) NPCs lysates were immunoprecipitated with anti-IgG, -Mbd3 conjugated beads and were analyzed by immunoblotting for indicated proteins. (H) HEK293 cells were transfected with empty or Smek1 expression plasmids. At 24 hours after transfection, lysates were immunoprecipitated with anti-IgG or anti-Mbd3 (n = 2) and were analyzed by immunoblotting for indicated proteins. The underlying data set for panels A, B, C, D, and F can be found in the S1 Data file and all individual quantification data for panels C and F can be found in S2 Data file.
(TIF)

S8 Fig Mbd3 gain-of-function in NPC neural differentiation. (A) NPCs transfected with mutant (ΔN92) Mbd3 were cultured under undifferentiated, U, and differentiated, D, conditions for 3 days, and indicated transcripts were analyzed by RT-PCR (n = 1). (B) NPCs were transfected with an Mbd3 expression vector and cultured under undifferentiated and differentiated conditions for 3 days. Indicated transcripts were analyzed by RT-PCR (n = 1). (C) NPCs were infected with Mbd3 lentiviral vector and one day later cultured under differentiation conditions for 5 days and then immunostained with Nestin/Mbd3/DAPI (upper row), NeuN/Mbd3/DAPI (middle row), or GFAP/Mbd3/DAPI (bottom row). Scale bars: 25 mm. (D) The effect of Mbd3 overexpression on NPC differentiation based on counting NeuN- or GFAP-positive cells among total Mbd3-positive cells. Bar graphs represent means ± S.D. (n = 3). OE, overexpression. *P < 0.05 (Student *t*-test). N.S, *P* > 0.05. The underlying quantification data for panel D can be found in the S2 Data file.
(TIF)

S9 Fig Effect of PP4c knockdown on neurogenesis and Smek-mediated Mbd3 protein stability, and effect of NuRD components inhibition on neurogenesis of NPCs. (A) *Wild-type* NPCs were transfected with either control siRNA or siPP4c and grown for 2 days in N2 medium with bFGF. qPCR analysis was performed to detect indicated mRNAs. (B) HEK293 cell lines stably overexpressing Smek1 were transfected with indicated constructs, treated with MG132 for 6 hours, and immunoprecipitated with myc-conjugated beads. Mbd3 ubiquitylation was detected by immunoblot with anti-HA antibody. Smek1, PP4c, and a-tubulin in lysates were detected by immunoblotting (n = 3). (C) *Smek1/2 dKO* NPCs were transfected with indicated siRNAs and grown for 2 days in N2 medium with bFGF. Indicated proteins were detected by immunoblotting. Relative (Rel.) Tuj1/a-tubulin was quantified by the Image J quantification software (n = 2). (D-F) *Smek1/2 dKO* NPCs were treated with indicated HDAC inhibitors and grown for 2 days in N2 medium with bFGF. (D) Indicated proteins in lysates were detected by immunoblotting and (E-F) qPCR analysis was performed to detect indicated mRNAs. The underlying all individual quantification data for panels A, E, and F can be found in the S2 Data File.
(TIF)

S10 Fig Putative functional link between *TRIM33* and *Mbd3* genes. (A) Trails indicate putative functional links between *TRIM3*3 (context) and *Mbd3* (target). (B) Function relationships were analyzed using Biograph software. In the context of *Mbd3* genes, TRIM33 ranks #15 of 18179 'Gene' concepts (top 0.08%).
(TIF)

S1 Table Comparison of neurogenesis defects seen in embryonic brain of *Smek1 ko* and *Smek1/2 dko* mice. (DOCX)

S2 Table Smek1 interacting proteins by yeast two-hybrid screening. (DOCX)

S3 Table GPS (group based prediction system) 3.0 software (http://gps.biocuckoo.org) based prediction of kinases for Mbd3 phosphorylation. (DOCX)

S4 Table Construction of pLKO3G shRNA and target sequences. (DOCX)

S5 Table Primer sequences used for quantitative or RT- PCR analysis. (DOCX)

S6 Table Primer sequences used for ChIP-qPCR. (DOCX)

S1 Data List of all individual quantitative data values that underlie the data summarized in the Smek1-ChIP sequencing. (TXT)

S2 Data All individual quantitative data that were used to generate graph and histogram in each figure. (XLSX)

## Abbreviations

2Diff: differentiation 2 d in vitro
5′-hmC: 5′-hydroxymethyl-cytosine
5′-mC: 5′-methyl-cytosine
Ac: acetylation
bFGF: basic fibroblast growth factor
ChIP-seq: chromatin immunoprecipitation sequencing
CHX: cycloheximide
CNS: central nervous system
CREB: cAMP-response element binding protein
CRTC2: CREB-regulated transcriptional coactivator 2
CP: cortical plate
DAPI: 4',6-diamidino-2-phenylindole
DIV: days in vitro
dKO: double knockout
EGFP: enhanced green fluorescent protein
ESC: embryonic stem cell
Fb: forebrain
GO: gene ontology
GST: glutathione-S-transferase
HA: human influenza hemagglutinin
IB: immunoblot
IgG: immunoglobulin G
IP: immunoprecipitation
IZ: intermediate zone
KD: knockdown
KO: knockout
MAP2: microtubule-associated protein 2
Mbd: methyl-CpG–binding domain
Mbd3: methyl-CpG–binding domain protein 3
NEC: neuroepithelial cell
NPC: neural progenitor cell
ns: not significant
NSC: neural stem cell
NuRD: nucleosome remodeling and deacetylase
OE: overexpression
PKC: protein kinase C
qPCR: quantitative PCR
RING: Really Interesting New Gene
RPC: lineage-restricted precursor cell
RT-PCR: reverse transcription PCR
Smek: Suppressor of Mek null
SVZ: subventricular zone
TRIM33: TRIpartite Motif protein 33
TSS: transcription starting site
Ub: ubiquitin
Un: undifferentiation
VZ: ventricular zone
WCL: whole cell lysate
WT: wild type
Y2H: yeast two hybrid
