# Supplementary figures and images for: Smek promotes corticogenesis through regulating Mbd3’s stability and Mbd3/NuRD complex recruitment to genes associated with neurogenesis
Source: PLoS Biol. 2017 May 3;15(5):e2001220. doi: 10.1371/journal.pbio.2001220 (PMC5414985; doi:10.1371/journal.pbio.2001220)

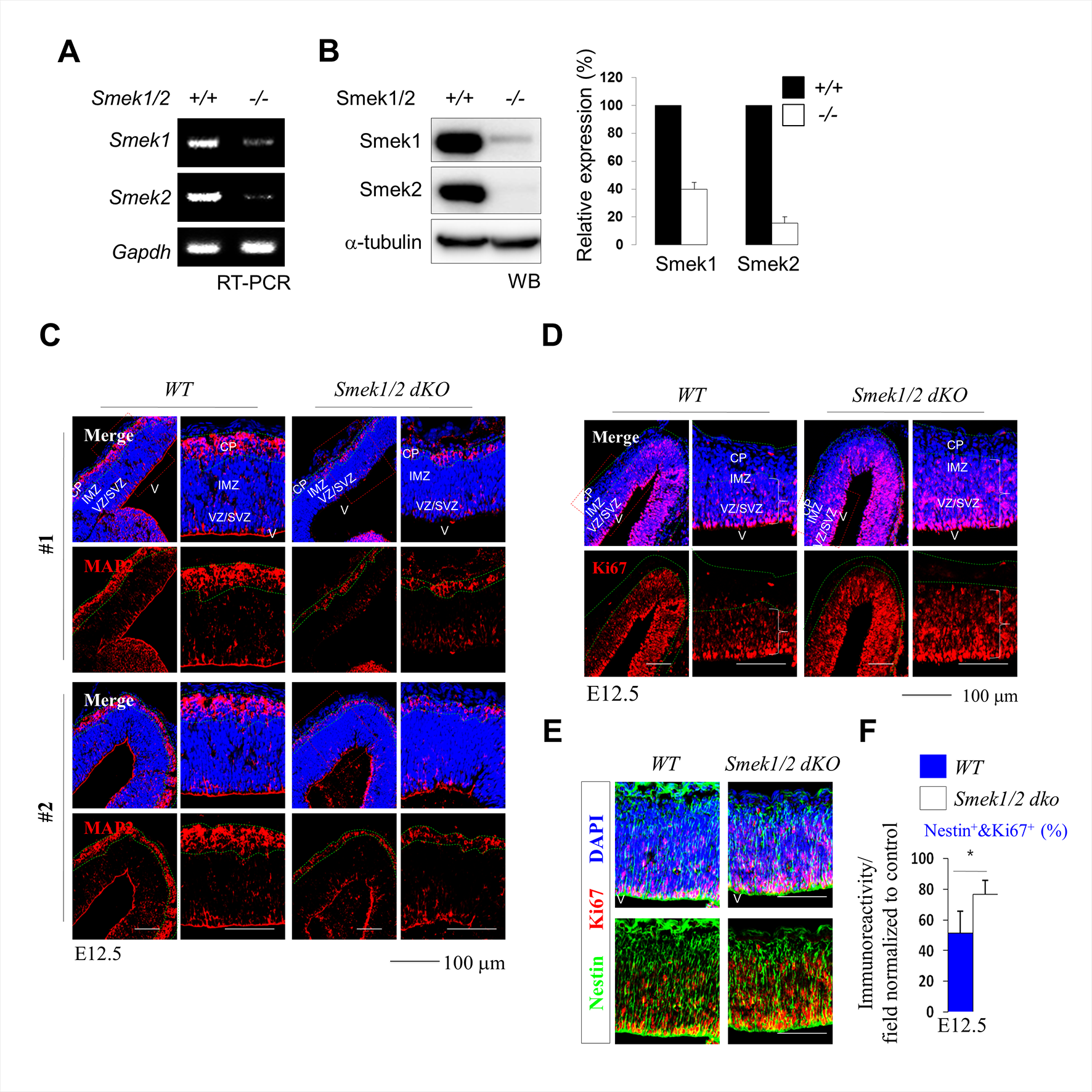

Supplement: S1 Fig — (A, B) Gene trapped mutant Smek1 and Smek2 ES cell (E14) were injected into mouse blastocyst and chimeric mice were backcrossed with C57BL/6 mice. After 6 generation, mice were used for analysis. (A) cDNA was prepared from total RNA harvested from wild-type and Smek1/2 dko NPCs and expression of indicated genes was measured by RT-PCR. (B) Respective lysates were immunoblotted with indicated antibodies (n = 2). Quantification of band intensity was done using Image J. (C) Representative images showing MAP2 staining at E12.5 (see Fig 1A, right panel). (D-F) Coronal sections from E12.5 mouse brain stained with antibodies against Ki67 (red), Nestin (green), and MAP2 (red). Nuclei were counterstained with DAPI (blue). Scale bar: 100 mm. (E) Identification of self-renewal and proliferating NPCs by Ki67 and Nestin staining in E12.5 mouse cortex (WT, n = 7; Smek1/2 dKO, n = 5). (F) Quantification of S1E Fig. Data is quantified using the Image J software. Bar graphs represent means ± S.D. *P < 0.05 (unpaired Student t-test). Scale bar: 100 mm. The underlying data for panels B and F can be found in the S2 Data file. (TIF) [file pbio.2001220.s001.TIF]

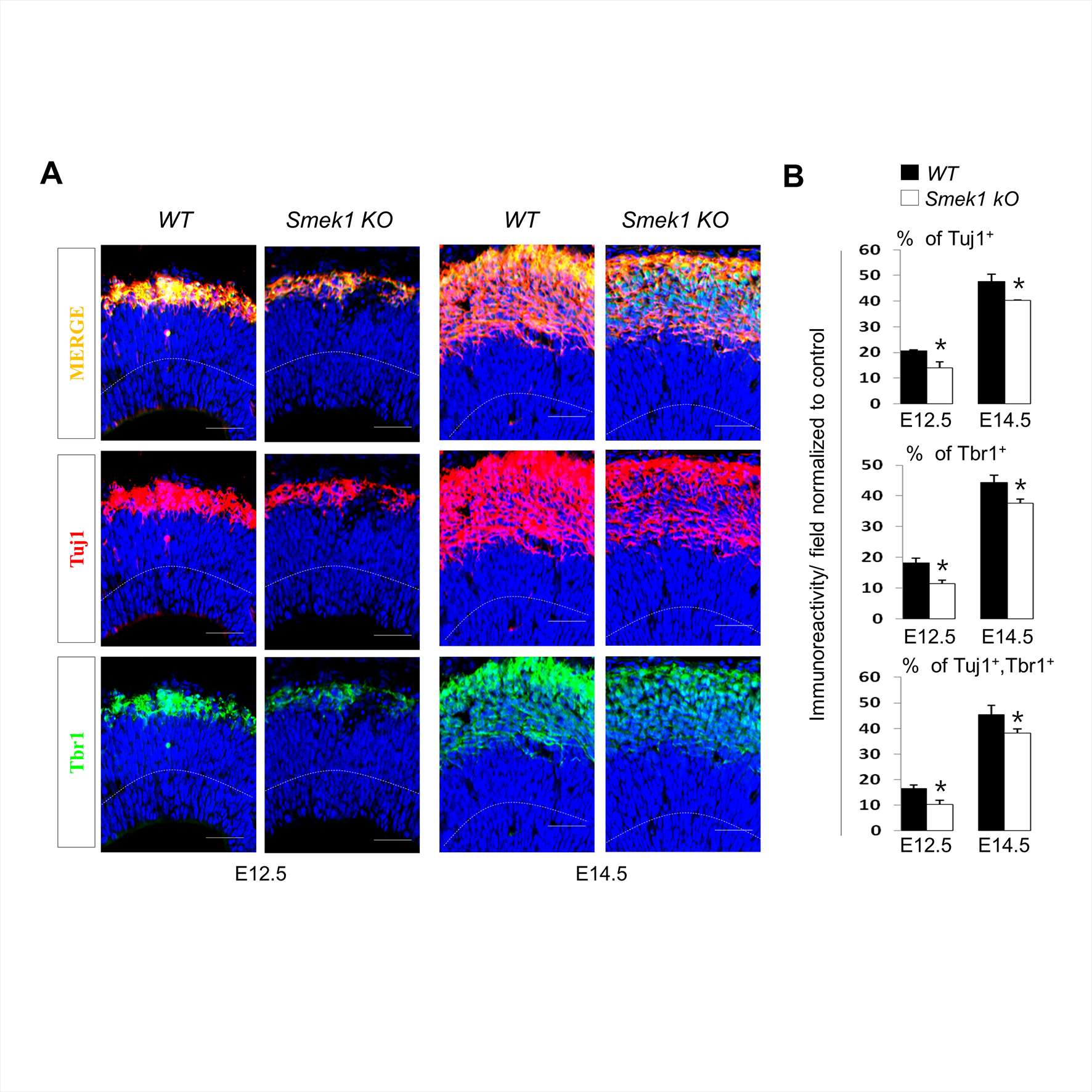

Supplement: S2 Fig — (A) Coronal sections from WT or Smek1 KO cortex at E12.5 or E14.5 were stained with Tuj1 (red) and Tbr1 (green) antibodies. Nuclei were counterstained with DAPI (blue). Scale bar: 100 mm. (B) Quantification of staining for Tuj1+, Tbr1+, or double-positive cells using the Image J software. Bar graphs represent means ± S.D. (n = 3). *P < 0.05 (Student’s t-test). The underlying data for panel B can be found in the S2 Data file. (TIF) [file pbio.2001220.s002.TIF]

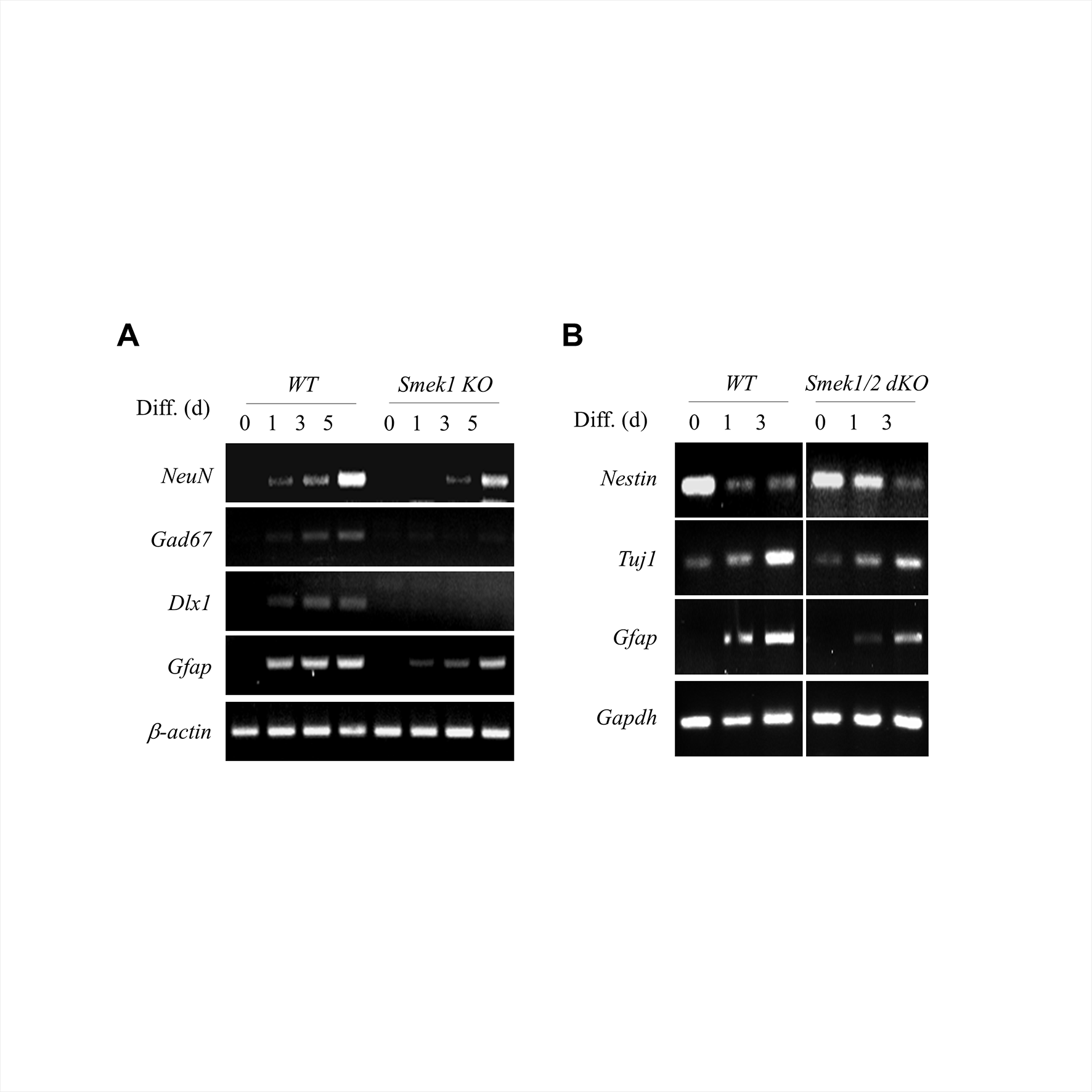

Supplement: S3 Fig — (A and B) Smek1 KO, Smek1/2 dKO or Wild-type NPCs were grown in N2 medium without bFGF for indicated days. cDNA was prepared from total RNA harvested from WT, Smek1KO and Smek1/2 dKO NPCs and expression of indicated genes was measured by RT-PCR (n = 2). Diff. (d), days in differentiation. (TIF) [file pbio.2001220.s003.TIF]

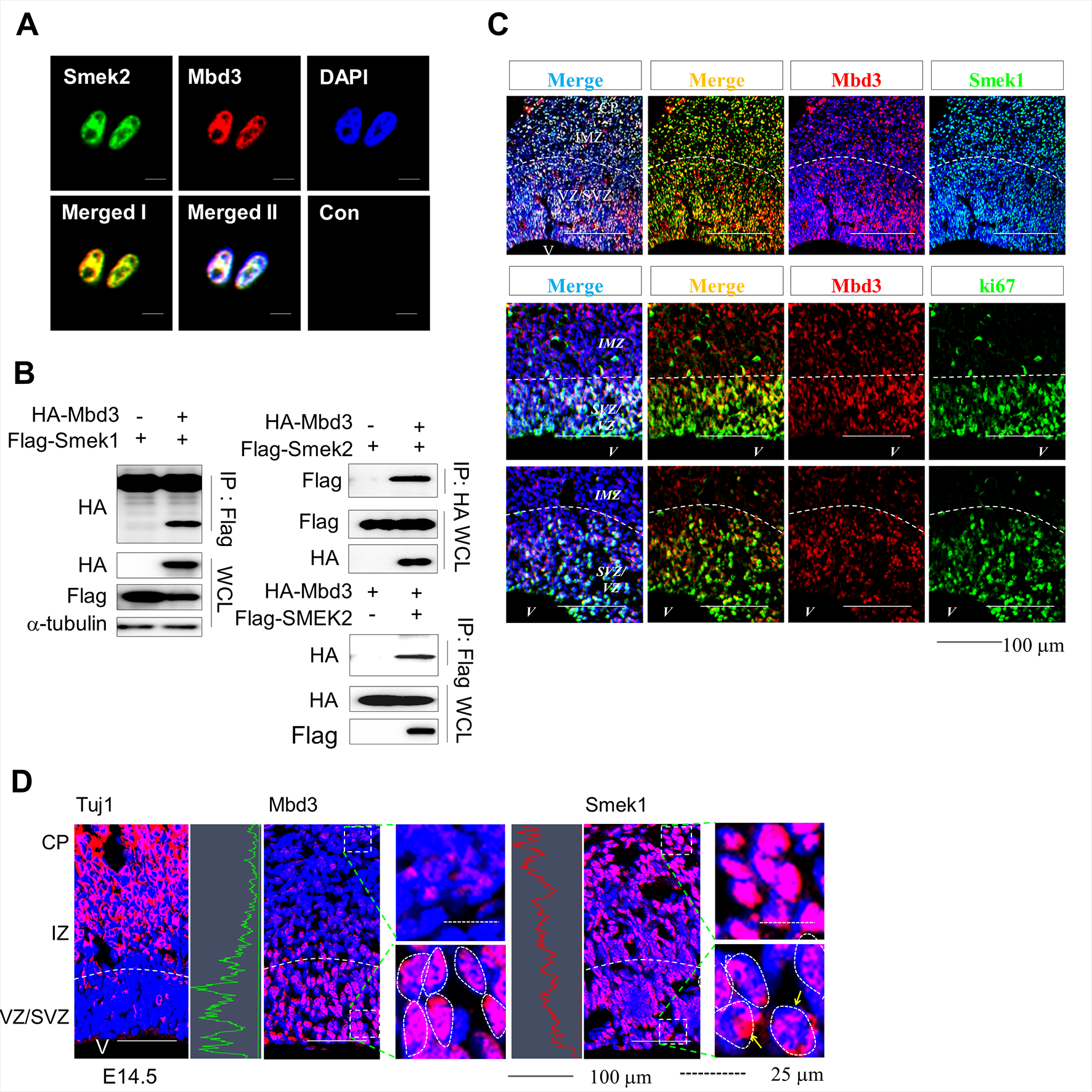

Supplement: S4 Fig — (A) Immunostaining with Mbd3 (red) and Smek2 (green) antibodies in HEK293 cells. DAPI (blue). Scale bars, 50 mm. (B) Immunoprecipitation (IP) using Flag or HA antibodies from lysates with either Flag-Smek1 or -Smek2 in the presence or absence of HA-Mbd3, or HA-Mbd3 plus control vector or Flag-Smek2 (n = 2). (C-D) Paraformaldehyde (PFA)-fixed, cyro-embedded coronal sections from E12.5 and E14.5 mouse cortex were stained with antibodies against Mbd3 (red), Smek1 (green or red) and Ki67 (green). Nuclei were counterstained with DAPI (blue). Yellow arrows indicate perinuclear localization of Smek1 in ventricular zone progenitor cells. Images were captured using a Zeiss confocal microscope. Scale bar: 25 or 100 mm. (D) Quantification of endogenous Mbd3 (green line) and Smek1 (red line) expression pattern was shown using the ZEN lite image software (http://www.zeiss.com/). (TIF) [file pbio.2001220.s004.TIF]

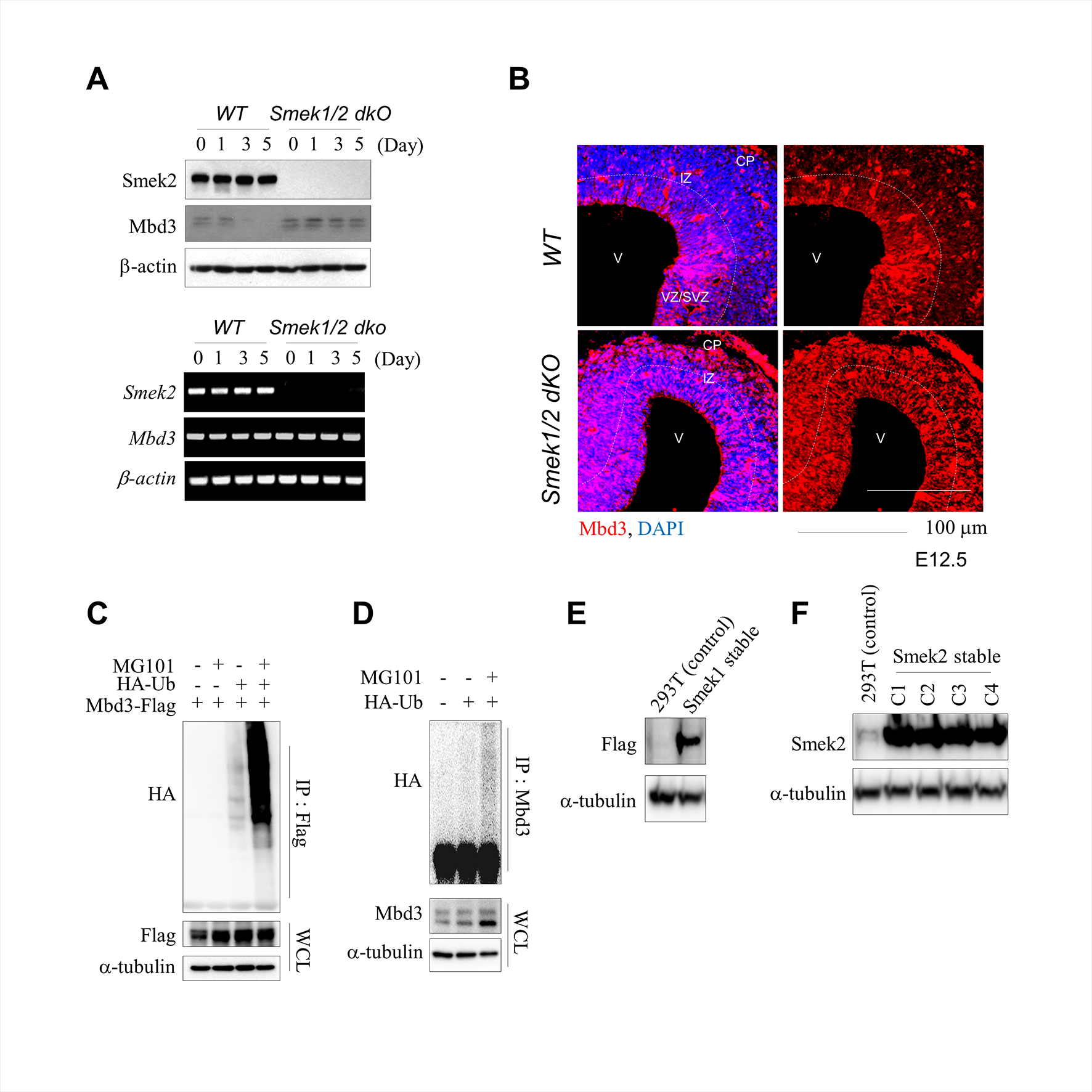

Supplement: S5 Fig — (A, upper panel) NPCs were grown in N2 medium without bFGF for indicated days, and lysates were immunoblotted with indicated antibodies (n = 2). (A, lower panel) cDNA was prepared from total RNA from Wild-type or Smek1/2 dKO NPCs, and indicated transcript levels were measured by RT-PCR (n = 2). (B) Paraformaldehyde (PFA)-fixed, cyro-embedded coronal sections from Wild-type or Smek1/2 dKO E12.5 mouse cortex were stained with antibodies against Mbd3 (red). Nuclei were counterstained with DAPI (blue). Images were captured using a Zeiss confocal microscope. Scale bars: 100 mm. (C) HEK293 cells were transfected with plasmids expressing Mbd3-Flag and HA-Ub, or Mbd3-Flag alone. At 24 hours after transfection, cells were treated with MG101 (25 μg/ml) for 12 hours before harvest. Lysates were prepared and immunoprecipitated using anti-Flag beads Mbd3 ubiquitylation was detected by immunoblotting with anti-HA antibody. Lysates were analyzed by immunoblotting for indicated proteins (n = 2). Ub, Ubiquitin. (D) Same as S5C Fig except using A/G beads incubated with anti-Mbd3 (n = 1). (E and F) HEK293 cells were infected with supernatants of lentivirus expressing Smek1 or Smek2. These cells were further selected during 2 weeks in medium contained puromycin (1ug/ml) and their expression was confirmed by immunoblotting with anti-Smek1 or anti-Smek2 antibodies (n = 2). (TIF) [file pbio.2001220.s005.TIF]

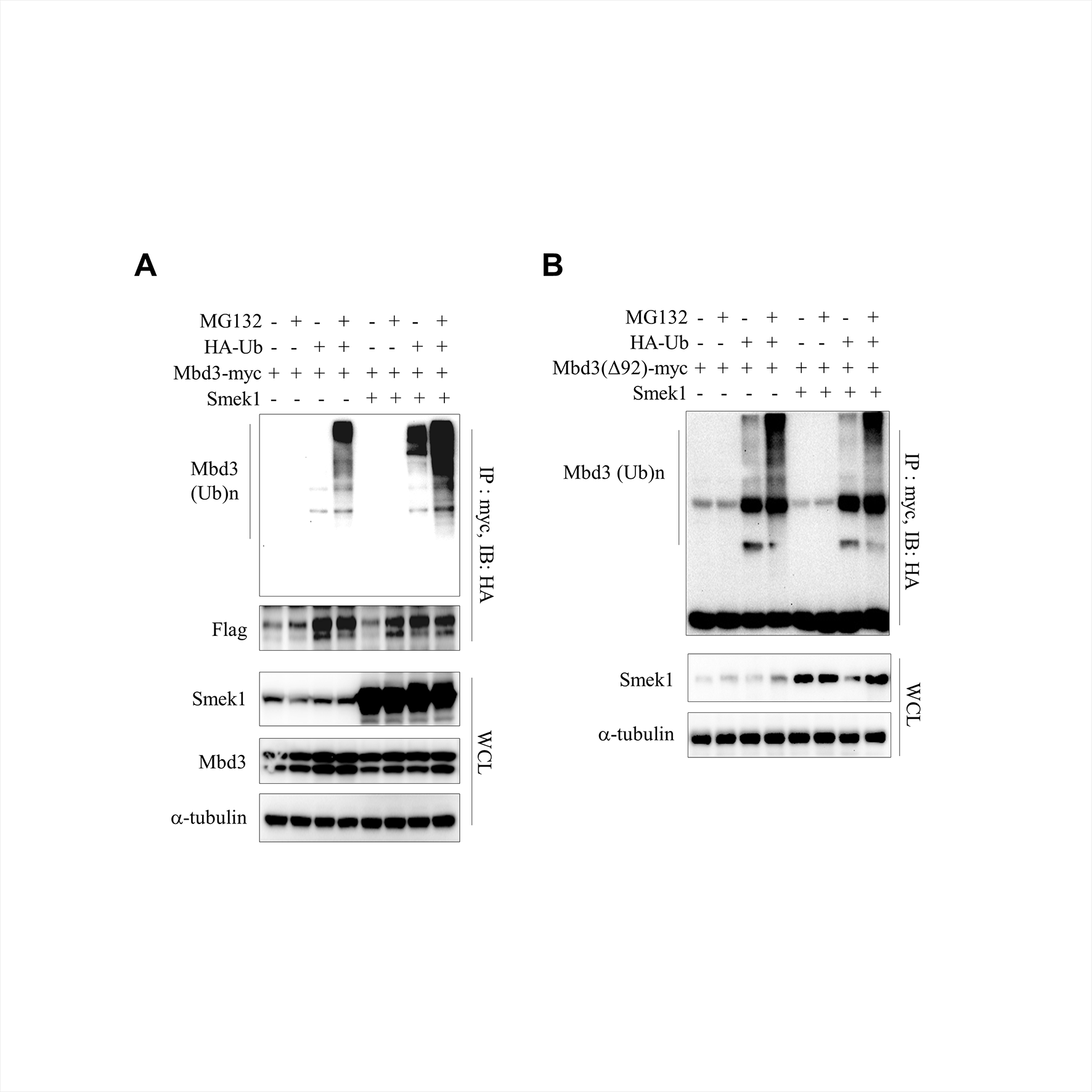

Supplement: S6 Fig — (A) HEK293 cells and lines stably overexpressing Smek1 were transfected with vector, Mbd3-Flag, and HA-Ub expression plasmids. One day later, cells were treated with MG132 for 6 hours, and lysates were immunoprecipitated with anti-myc beads (n = 4). (B) HEK293 cells and lines stably overexpressing Smek1 were transfected with indicated constructs, treated with MG132 for 6 hours, and immunoprecipitated with myc-conjugated beads. Mbd3 ubiquitylation was detected by immunoblot with anti-HA antibody. Smek1, Mbd3, and a-tubulin in lysates were detected by immunoblotting (n = 2). (TIF) [file pbio.2001220.s006.TIF]

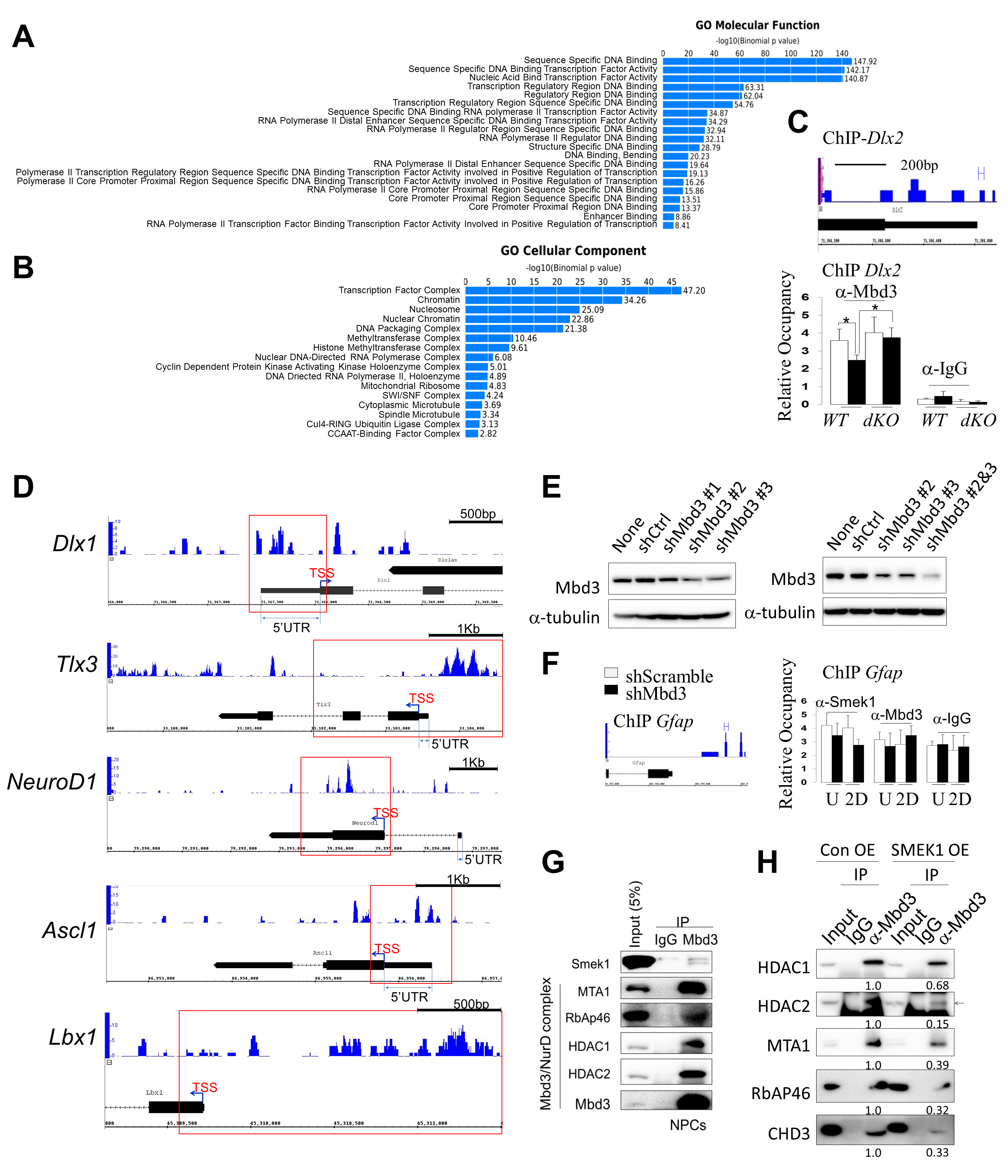

Supplement: S7 Fig — (A) Molecular function based on Gene ontology (GO) analysis. (B) Cellular function based on Gene ontology (GO) analysis. (C) (upper panel) Smek1 binding peaks in NPCs in differentiation genes such as Dlx2. Scale bar, 200 bp. (Lower panel) ChIP-qPCR analysis of Mbd3 occupancy at a Smek binding loci (Dlx2 gene promoter region) in undifferentiated or differentiated conditions in WT (n = 3) and Smek1/2 dKO (n = 3) NPCs. IgG ChIP served as a negative controls. (D) Smek1 binding peaks in NPCs in differentiation genes such as Dlx1, Tlx3, NeuroD1, Ascl1, and Lbx1. (E) NPCs were transfected with pLKO3G-shMbd3 #1, #2, and #3, and one day later, Mbd3 and a-tubulin in lysates were detected by immunoblotting (n = 2). (F) ChIP-qPCR analysis of Smek1 and Mbd3 occupancy at a Smek binding locus (Gfap gene promoter) in undifferentiated or differentiated conditions in NPCs knock-downed by shscramble (n = 3) and shMbd3 (n = 3) NPCs. IgG ChIP served as a negative control. Values are normalized to input control and represent average ±SD. t-test analysis was performed to calculate the statistical significance (*P < 0.05, **P < 0.005). (G) NPCs lysates were immunoprecipitated with anti-IgG, -Mbd3 conjugated beads and were analyzed by immunoblotting for indicated proteins. (H) HEK293 cells were transfected with empty or Smek1 expression plasmids. At 24 hours after transfection, lysates were immunoprecipitated with anti-IgG or anti-Mbd3 (n = 2) and were analyzed by immunoblotting for indicated proteins. The underlying data set for panels A, B, C, D, and F can be found in the S1 Data file and all individual quantification data for panels C and F can be found in S2 Data file. (TIF) [file pbio.2001220.s007.tif]

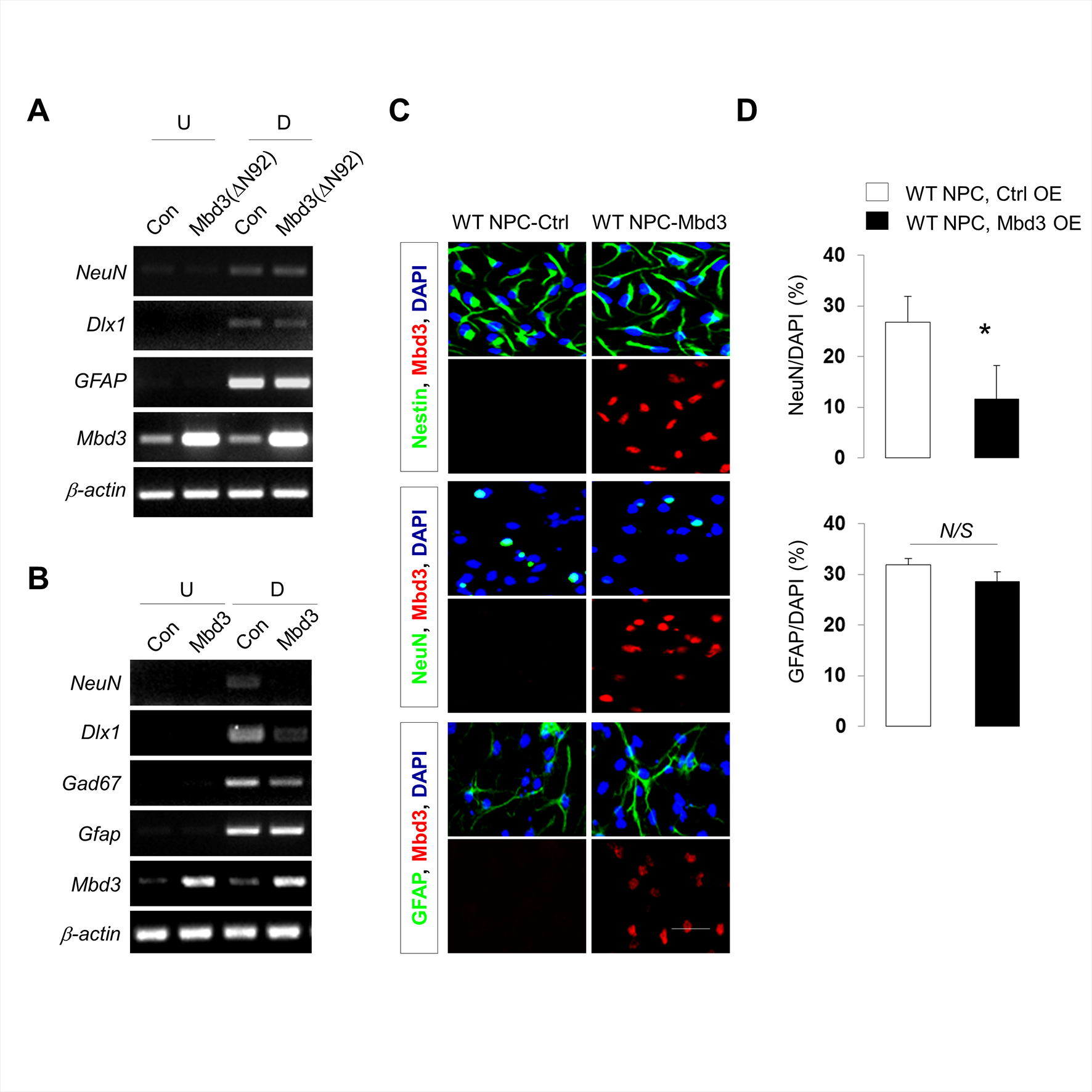

Supplement: S8 Fig — (A) NPCs transfected with mutant (ΔN92) Mbd3 were cultured under undifferentiated, U, and differentiated, D, conditions for 3 days, and indicated transcripts were analyzed by RT-PCR (n = 1). (B) NPCs were transfected with an Mbd3 expression vector and cultured under undifferentiated and differentiated conditions for 3 days. Indicated transcripts were analyzed by RT-PCR (n = 1). (C) NPCs were infected with Mbd3 lentiviral vector and one day later cultured under differentiation conditions for 5 days and then immunostained with Nestin/Mbd3/DAPI (upper row), NeuN/Mbd3/DAPI (middle row), or GFAP/Mbd3/DAPI (bottom row). Scale bars: 25 mm. (D) The effect of Mbd3 overexpression on NPC differentiation based on counting NeuN- or GFAP-positive cells among total Mbd3-positive cells. Bar graphs represent means ± S.D. (n = 3). OE, overexpression. *P < 0.05 (Student t-test). N.S, P > 0.05. The underlying quantification data for panel D can be found in the S2 Data file. (TIF) [file pbio.2001220.s008.TIF]

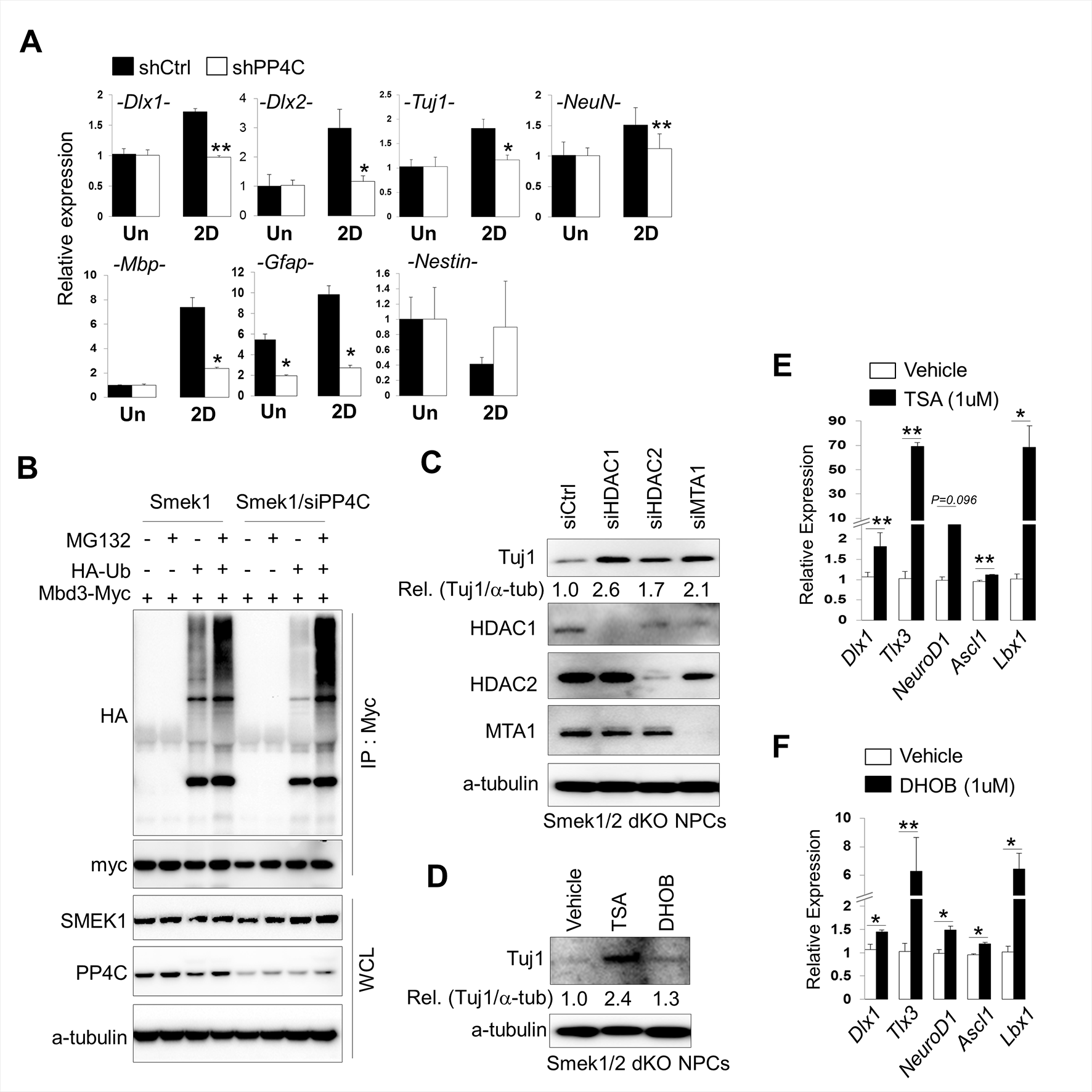

Supplement: S9 Fig — (A) Wild-type NPCs were transfected with either control siRNA or siPP4c and grown for 2 days in N2 medium with bFGF. qPCR analysis was performed to detect indicated mRNAs. (B) HEK293 cell lines stably overexpressing Smek1 were transfected with indicated constructs, treated with MG132 for 6 hours, and immunoprecipitated with myc-conjugated beads. Mbd3 ubiquitylation was detected by immunoblot with anti-HA antibody. Smek1, PP4c, and a-tubulin in lysates were detected by immunoblotting (n = 3). (C) Smek1/2 dKO NPCs were transfected with indicated siRNAs and grown for 2 days in N2 medium with bFGF. Indicated proteins were detected by immunoblotting. Relative (Rel.) Tuj1/a-tubulin was quantified by the Image J quantification software (n = 2). (D-F) Smek1/2 dKO NPCs were treated with indicated HDAC inhibitors and grown for 2 days in N2 medium with bFGF. (D) Indicated proteins in lysates were detected by immunoblotting and (E-F) qPCR analysis was performed to detect indicated mRNAs. The underlying all individual quantification data for panels A, E, and F can be found in the S2 Data File. (TIF) [file pbio.2001220.s009.TIF]

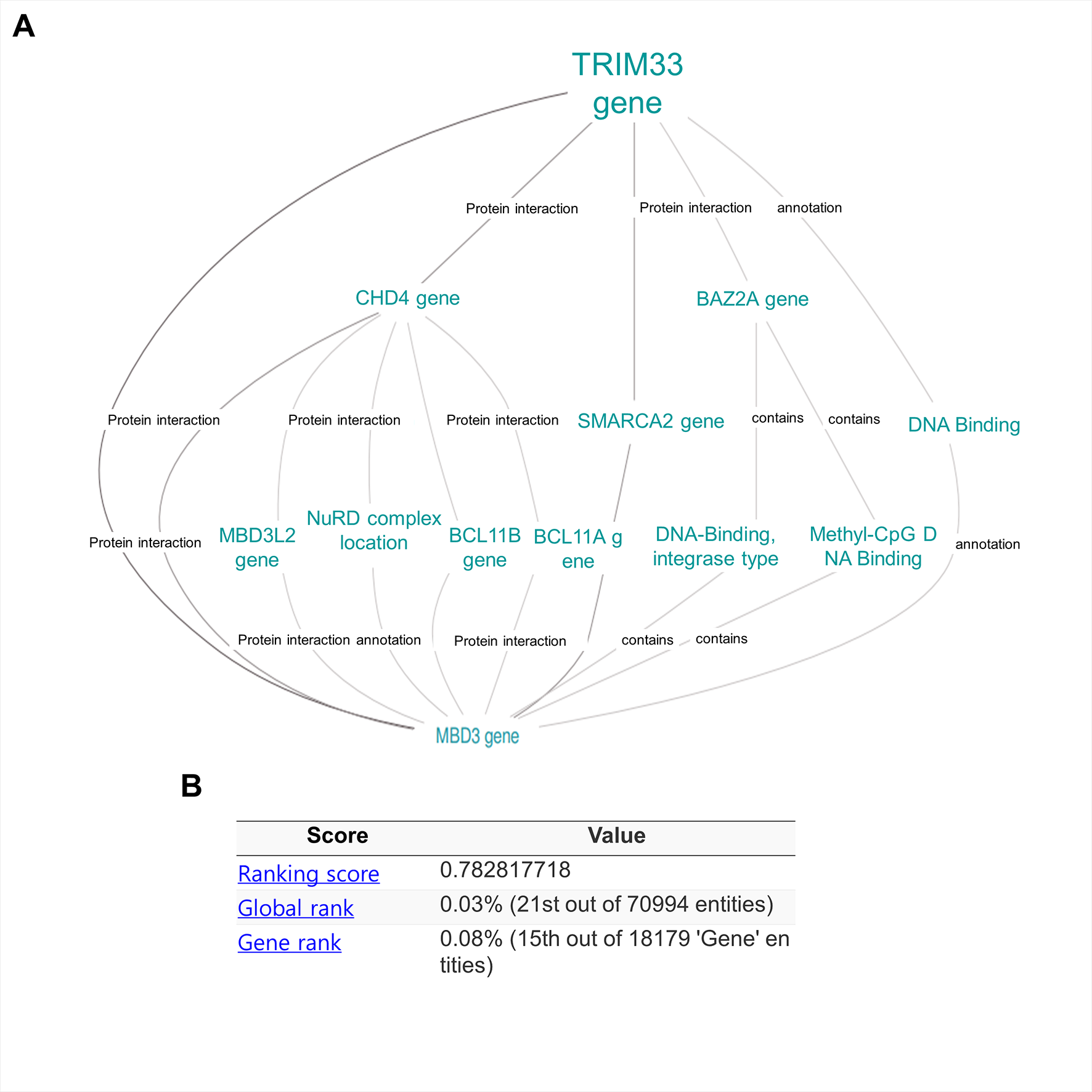

Supplement: S10 Fig — (A) Trails indicate putative functional links between TRIM33 (context) and Mbd3 (target). (B) Function relationships were analyzed using Biograph software. In the context of Mbd3 genes, TRIM33 ranks #15 of 18179 'Gene' concepts (top 0.08%). (TIF) [file pbio.2001220.s010.TIF]

**Supporting Information**

**S6 Table. Primer sequences used for ChIP-qPCR.**

**
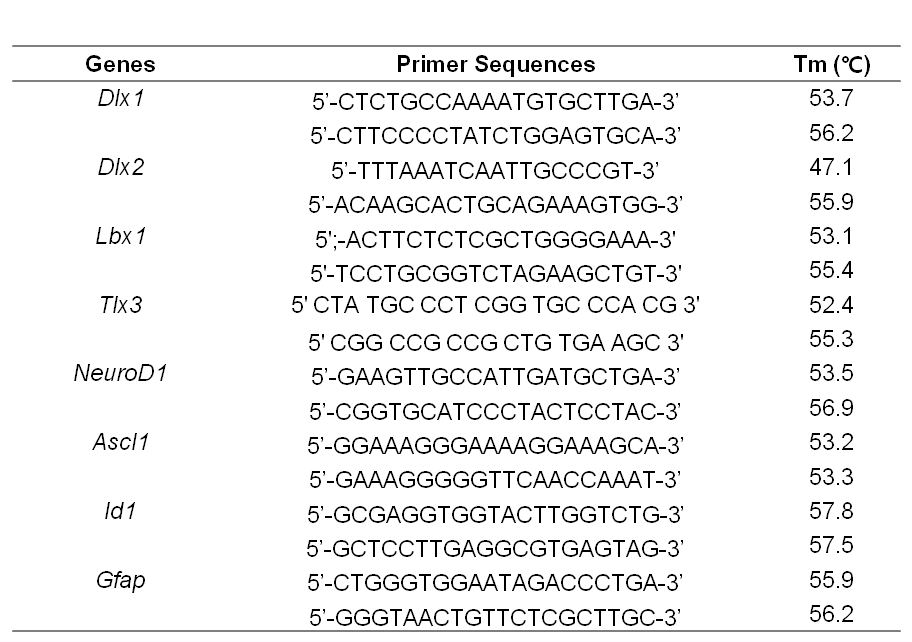
**

Supplement: S6 Table — (DOCX) [file pbio.2001220.s016.docx]
