# Supplementary material for: Smek promotes corticogenesis through regulating Mbd3’s stability and Mbd3/NuRD complex recruitment to genes associated with neurogenesis
Source: PLoS Biol. 2017 May 3;15(5):e2001220. doi: 10.1371/journal.pbio.2001220 (PMC5414985; doi:10.1371/journal.pbio.2001220)
Supplement: S1 Table — (DOCX) [file pbio.2001220.s011.docx]

**Supporting Information**

**S1 Table. Comparison of neurogenesis defects seen in embryonic brain of *Smek1 ko* and *Smek1/2 dko* mice.**

|  |  | **Tuj1+ cells /% decrease** | **Tbr1+ cells /% decrease** | **Tuj1+&Tbr1+cells /% decrease** |
| --- | --- | --- | --- | --- |
| E12.5 | WT vs SMEK1 KO | 6% | 6.60% | 6.20% |
| E14.5 | WT vs SMEK1 KO | 7% | 6.60% | 6.30% |
|  |  |  |  |  |
| E12.5 | WT vs SMEK dKO | 13% | 15% | 20% |
| E14.5 | WT vs SMEK dKO | 24% | 20% | 20% |
