# Supplementary material for: Smek promotes corticogenesis through regulating Mbd3’s stability and Mbd3/NuRD complex recruitment to genes associated with neurogenesis
Source: PLoS Biol. 2017 May 3;15(5):e2001220. doi: 10.1371/journal.pbio.2001220 (PMC5414985; doi:10.1371/journal.pbio.2001220)
Supplement: S2 Table — (DOCX) [file pbio.2001220.s012.docx]

**Supporting Information**

**S2 Table. Smek1 interacting proteins by yeast two hybrid screening.**

| **Gene name** | **Number of clones** | **Lac Z Bait alone** | **LacZ Bait+Prey** |
| --- | --- | --- | --- |
| DNMT1 associated protein | 27 | - | + |
| Ski interacting protein | 8 | - | +++ |
| Cdca8 | 8 | - | +++ |
| Rex2, RNA exonuclease2 homologue | 8 | - | + |
| Methyl-CpG binding domain protein 3 (Mbd3) | 6 | - | + |
| Era (G protein) like 1 | 5 | - | +++ |
