# Supplementary material for: Smek promotes corticogenesis through regulating Mbd3’s stability and Mbd3/NuRD complex recruitment to genes associated with neurogenesis
Source: PLoS Biol. 2017 May 3;15(5):e2001220. doi: 10.1371/journal.pbio.2001220 (PMC5414985; doi:10.1371/journal.pbio.2001220)

**Supporting Information**

**S3 Table. GPS (group based prediction system) 3.0 software (**[**http://gps.biocuckoo.org**](http://gps.biocuckoo.org)**) based prediction of kinases for Mbd3 phosphorylation .**


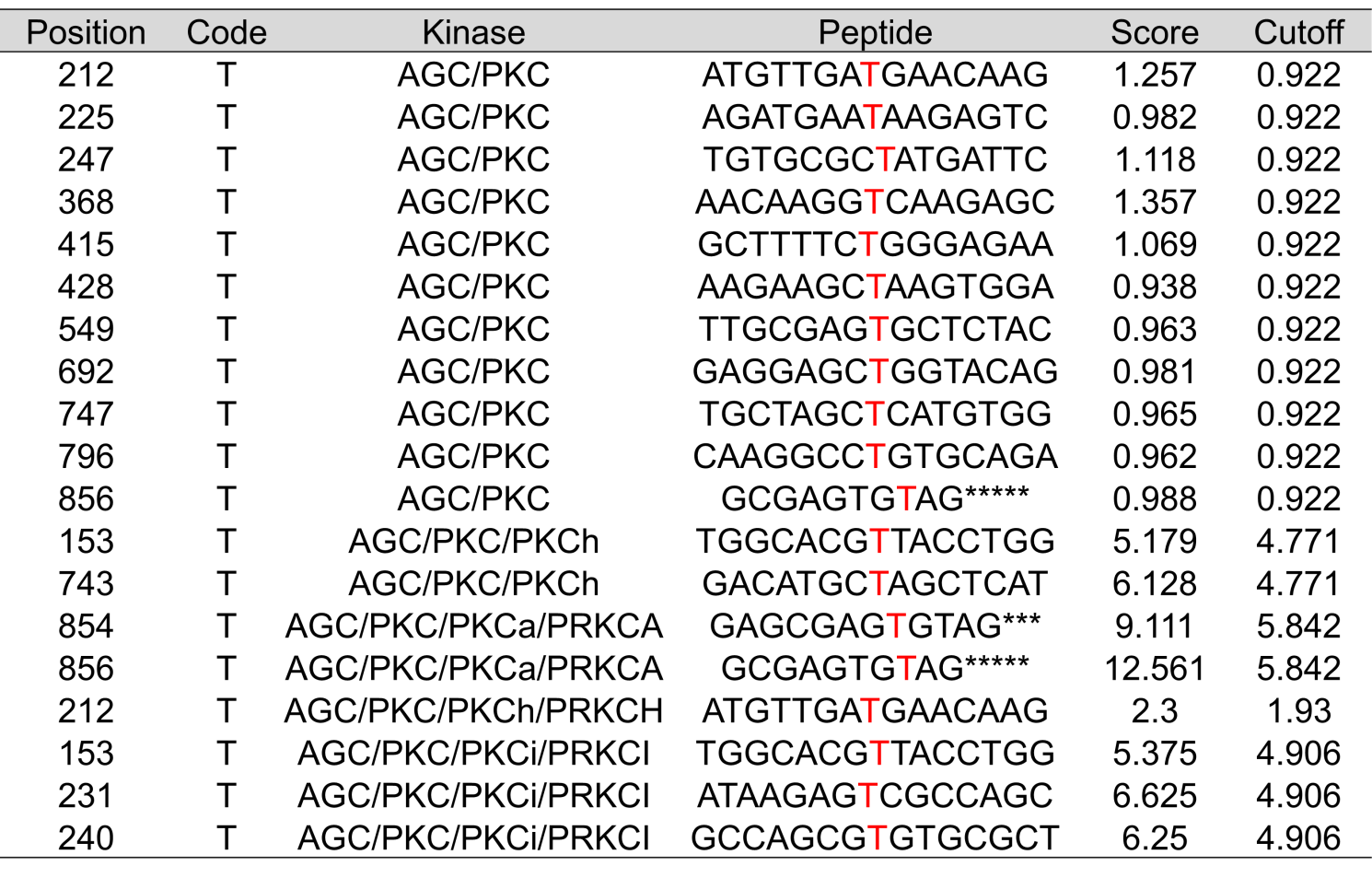

Supplement: S3 Table — (DOCX) [file pbio.2001220.s013.docx]
