# Supplementary material for: Smek promotes corticogenesis through regulating Mbd3’s stability and Mbd3/NuRD complex recruitment to genes associated with neurogenesis
Source: PLoS Biol. 2017 May 3;15(5):e2001220. doi: 10.1371/journal.pbio.2001220 (PMC5414985; doi:10.1371/journal.pbio.2001220)

**Supporting Information**

**S4 Table. Construction of pLKO3G shRNA and target sequences.**

To knock down Mbd3 expression in NSCs we generated **pLKO.3G** short hairpin (sh)RNA lenti-viral vector system. For Mbd3, target sequences were chosen using RNAi consortium shRNA library (<http://www.broadinstitute.org/rnai/public/>) (three clones targeting different sequences in the two coding regions and one 3’UTR of Mbd3 gene) against Mbd3. To generate oligos for cloning, sense and antisense sequences of chosen target sequences were ordered from IDT. Sequence-verified shRNA lentiviral plasmid vectors for mouse Mbd3 gene were cloned into the pLKO.3G vector and knockdown efficiency were measured by western blot (see S7E Fig).

For gene transfections we utilized Lipofectamine 3000 and Amaxa nucleofection (Lonza, http: //www.lonzabio.com/cell-biology/transfection/) for the transfection of mouse NSCs. A range of knockdown efficiencies was observed. One (we encoded it as shMbd3 #3) out of three clones appeared to be efficiently knocking down Mbd3 expression in NSCs (see S7E Fig). The shMbd3 (#2 and #3) clones showed maximum efficiencies and both were used for further experiments. For shControl transfection, sequences were chosen based on Sigma-Aldrich shRNA products which had been previously validated as a non-target shRNA control. The plasmids containing the below sequences that does not target any gene, making it useful as a negative control in our experiments and appear no change Mbd3 expression (see S7E Fig).

.
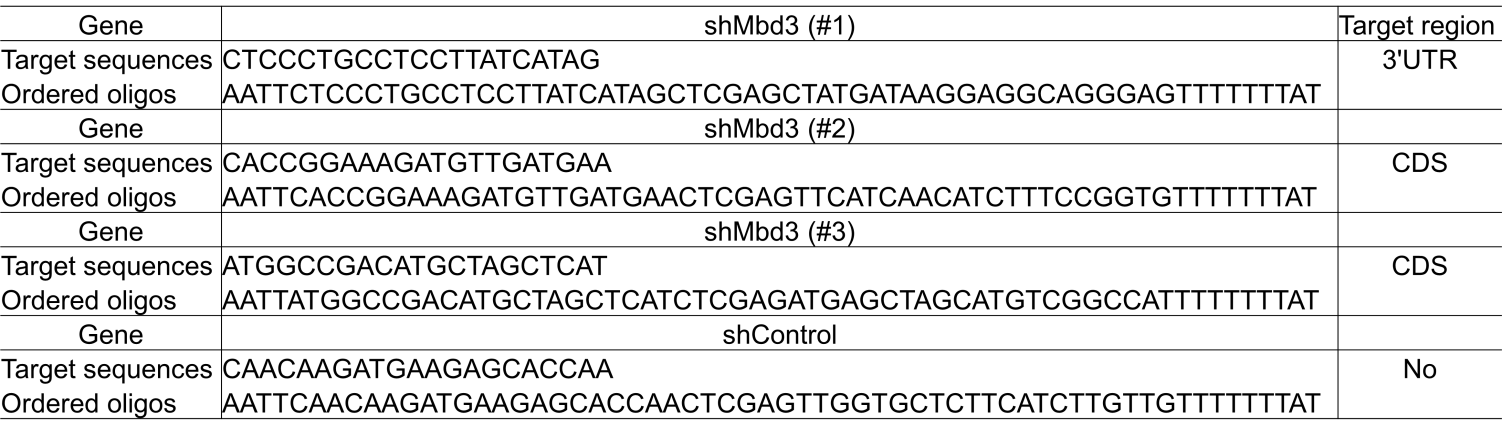

Supplement: S4 Table — (DOCX) [file pbio.2001220.s014.docx]
