# Supplementary material for: Smek promotes corticogenesis through regulating Mbd3’s stability and Mbd3/NuRD complex recruitment to genes associated with neurogenesis
Source: PLoS Biol. 2017 May 3;15(5):e2001220. doi: 10.1371/journal.pbio.2001220 (PMC5414985; doi:10.1371/journal.pbio.2001220)
Supplement: S5 Table — (DOCX) [file pbio.2001220.s015.docx]

**Supporting Information**

**S5 Table. Primer sequences used for quantitative or RT- PCR analysis.**

| **Genes** | **Primer Sequences** | **Tm (℃)** |
| --- | --- | --- |
| *Nestin* | 5’-CCCTGAAGTCGAGGAGCTG-3’ | 57.4 |
|  | 5’-CTGCTGCACCTCTAAGCGA-3’ | 57.3 |
| *Dlx1* | 5’-ATGCCAGAAAGTCTCAACAGC-3’ | 60.6 |
|  | 5’-AACAGTGCATGGAGTAGTGCC-3’ | 62.4 |
| *Dlx2* | 5’-AAAGAAAGTCCGGAAACCACG-3’ | 60.1 |
|  | 5’-TCTTCTTGAACTTGCATCGGC-3’ | 60.5 |
| *Tuj1* | 5’-TAGACCCCAGCGGCAACTAT-3’ | 58.2 |
|  | 5’-GTTCCAGGTTCCAAGTCCACC-3’ | 58.0 |
| *Gad67* | 5’-GCCACAAACTCAGCGGCATAGAAA-3’ | 60.0 |
|  | 5’-AGACGTCATACTGCTTGTCTGGCT-3’ | 60.0 |
| *Map2* | 5’-GCCAGCCTCAGAACAAACAG-3’ | 56.5 |
|  | 5’-AAGGTCTTGGGAGGGAAGAAC-3’ | 56.6 |
| *NeuN* | 5’-GAAACCGCAAGCCCTCATTTC-3’ | 60.1 |
|  | 5’-TTGGATGCCTCTTGGTTTGGT-3’ | 60 |
| *Gfap* | 5’-CCCTGGCTCGTGTGGATTT-3’ | 57.8 |
|  | 5’-GACCGATACCACTCCTCTGTC-3’ | 56.5 |
| *Mbp* | 5’-AATCGGCTCACAAGGGATTCA-3’ | 56.6 |
|  | 5’-TCCTCCCAGCTTAAAGATTTTGG-3’ | 55.1 |
| *SMEK1* | 5’-TATGACTTGGCCCTTAGCTTTCA-3’ | 56.2 |
|  | 5’-ACCTGGTGAGGACATATCATCA-3’ | 55.2 |
| *SMEK2* | 5’-AGGCGAGTGAAGGTCTATACC-3’ | 55.8 |
|  | 5’-GAGTAGCGATCCGTCGGACT-3’ | 58.4 |
| *Mbd3* | 5’-CCCCAGCGGGAAGAAGTTC-3’ | 58.4 |
|  | 5’-CGGAAGTCGAAGGTGCTGAG-3’ | 57.9 |
| *Gapdh* | 5’-AGGTCGGTGTGAACGGATTTG-3’ | 57.6 |
|  | 5’-TGTAGACCATGTAGTTGAGGTCA-3’ | 55.1 |
